# Public and animal health risks associated with spillover of *Brucella melitensis* into dairy farms

**DOI:** 10.1099/mgen.0.001014

**Published:** 2023-04-28

**Authors:** Svetlana Bardenstein, Daniel Grupel, Shlomo E. Blum, Yair Motro, Jacob Moran-Gilad

**Affiliations:** ^1^​ Kimron Veterinary Institute, Ministry of Agriculture and Rural Development, Beit Dagan, Israel; ^2^​ Internal Medicine B, Soroka University Medical Center, Beer Sheva, Israel; ^3^​ Department of Health Policy and Management, School of Public Health, Faculty of Health Sciences, Ben Gurion University of the Negev, Beer Sheva, Israel

**Keywords:** *Brucella melitensis*, cattle, Israel, one health, public health, genomics, epidemiology

## Abstract

Brucellosis is a worldwide zoonosis with important public health, animal health and economic implications. *Brucella melitensis *, commonly associated with small ruminants, is an emerging bovine pathogen in dairy farms. We analysed all *B. melitensis* outbreaks affecting dairy farms in Israel since 2006, combining traditional and genomic epidemiology to explore the public health implications of this One Health challenge. Whole-genome sequencing was applied to bovine and related human *B. melitensis* isolates from dairy farm outbreaks. cgMLST-based and SNP-based typing was integrated with epidemiological and investigation data. A secondary analysis combining the bovine-human isolates with endemic human isolates from southern Israel was performed. A total of 92 isolates from dairy cows and related human cases originating from 18 epidemiological clusters were analysed. Most genomic and epi-clusters were congruent, but sequencing showed relatedness between apparently unrelated farm outbreaks. Nine secondary human infections were also genomically confirmed. The bovine-human cohort appeared intermixed with 126 endemic human isolates in southern Israel. We show a persistent and widespread circulation of *B. melitensis* in dairy farms in Israel with secondary occupational human infection. The genomic epidemiology also uncovered cryptic connections between outbreaks. A regional connection between bovine and endemic human brucellosis cases points to a common reservoir, most probably local small ruminant herds. Control of humans and bovine brucellosis is inseparable. Epidemiological and microbiological surveillance and implementation of control measures across the entire range of farm animals is needed to mitigate this public health challenge.

## Data Summary

Sequencing read data of *B. melitensis* isolates have been deposited ENA at EMBL-EBI under accession number PRJEB52526 (https://www.ebi.ac.uk/ena/browser/view/PRJEB52526) and made public. Accession numbers of the individual samples are listed in Table S5 in the supplementary material (available in the online version of this article). *In silico* multilocus sequence typing (MLST) data of genomic sequences used, are summarised in Table S3. The sequence data were also submitted to PubMLST. The authors confirm that all supporting data and protocols have been provided within the article or through supplementary data files.

**Impact Statement**

Our study features a retrospective countrywide genomic analysis of *Brucella melitensis* clusters in dairy farms in Israel since 2006. Israel is endemic for *B. melitensis* in small ruminant herds (ovine and caprine) with subsequent human infections. Our genomic epidemiology analysis shows an unusual persistent and widespread circulation of *B. melitensis* in dairy farms in Israel with secondary occupational human infection and uncovered cryptic transmission events. Our findings suggest a spillover from endemic brucellosis into dairy farms, a risk that needs to be acknowledged in other endemic settings. Our findings also imply that the control of humans and animal is inseparable and should employ a One Health approach.

## Introduction

Brucellosis is a global zoonotic disease, causing a significant health burden, with over 500 000 cases reported annually worldwide [1]. While the mortality associated with brucellosis is relatively low, acute and chronic morbidity causes a substantial impact on patients and healthcare systems [2]. Brucellosis also significantly impacts animal health and production, due to the need for rigid control measures (i.e. culling of animals) and increased abortion rates [3–5].

Several species of the genus *Brucella* may cause brucellosis. While the species most commonly affecting animals worldwide is *B. abortus *, human infection is primarily caused by *B. melitensis *, followed by *B. abortus *, *B. suis* and *B. canis *. The main reservoir of *B. melitensis* is small ruminants, mainly sheep and goats. Human infection is mainly associated with the consumption of unpasteurised dairy products [6] and, to a lesser extent, occupational exposure among animal handlers and veterinarians. Other ruminants are occasionally implicated in human brucellosis, for example, dromedary camels [7].

In Israel, all forms of brucellosis are notifiable by law since 1951. Vaccination of cattle with the S19 vaccine and tight regulation over the dairy industry led to the elimination of *B. abortus* in 1984 [8].

*B. melitensis* is endemic to Israel, with a fluctuating incidence over the last decades, despite control efforts and Rev1 vaccination of small ruminants. Over the recent years, an increasing incidence of *B. melitensis* has been reported, disproportionately affecting the Arab population in Israel, and specifically the Bedouin Arab population in Southern Israel, having an incidence rate of up to 100-fold as compared to the Jewish population [9].

While *B. melitensis* is mainly limited to small ruminant herds, there have been sporadic reports of isolation of *B. melitensis* from cattle since 2000 [10–12]. Similarly, active surveillance in dairy farms in Israel demonstrates increasing reports of bovine infections with *B. melitensis *, which warrants further investigation due to the public health and veterinary consequences. In that respect, the recent development of typing methods based on whole-genome sequencing (WGS) [13] allows an in-depth investigation of the origin and subsequent spread of *Brucella* spp. in endemic and non-endemic settings [7, 14–17], thus allowing a unique opportunity to study brucellosis in a truly One Health context.

The current study retrospectively investigated epidemiological clusters of brucellosis affecting dairy farms in Israel (2006 to 2021). Our goals were to study the genomic epidemiology of *B. melitensis* infecting dairy farms, to elucidate the links between bovine and human *B. melitensis* infections, and to study these strains in the broader genomic context of potential spillover of circulating regional strains into dairy farms.

## Methods

### Study setting

The Israeli dairy farming industry is comprised of ca. 800 farms housing 135 000 milking cows. The farms operate under strict veterinary supervision in accordance with regulations set by the Israeli Ministry of Agriculture and Rural Development [18]. Each farm is assigned a level of brucellosis risk; those with a history of brucellosis (in the preceding ten years) or residing near infected small ruminant herds are considered high risk. All dairy farms conduct and report surveillance for abortions. Serological tests for brucellosis are mandatory following abortion episodes in cattle and prior to animal trade between farms. Full-herd serological screening is triggered according to the level of risk, and positive findings.

Milk, abortion material and blood samples are all sent for laboratory confirmation to the Israeli Brucellosis National Reference Laboratory at the Kimron Veterinary Institute, Beit Dagan, Israel.

### Study isolates and case selection

We retrospectively identified cases of *B. melitensis* infection in dairy farms and related epidemiological clusters occurring between 2006–2021, based on the Israeli Brucellosis National Reference Laboratory records. We retrieved basic epidemiological data available at the laboratory as part of routine outbreak investigations performed on every brucellosis case and *B. melitensis* isolates from the laboratory’s culture collection.

Isolates included in the study originated from the following sources (Tables S1 and S2):

- Veterinary clinical samples of aborted fetal tissue, milk or blood obtained during the investigation of suspected brucellosis in dairy farms. The triggers for sampling were either cases of late abortion among cattle or incident human brucellosis cases suggesting potential exposure to dairy cows.
- Veterinary blood cultures obtained following positive serological screening (using the serum complement fixation test [CFT] for *Brucella* IgG) performed per regulations upon sale or movement of cattle between farms or during contact tracing among animals around proven brucellosis cases. In cases of abortions, both CFT for IgG and serum agglutination for *Brucella* IgM are performed.
- Human clinical samples obtained as part of the clinical workup of suspected cases of brucellosis; suspected isolates are referred from respective hospitals throughout the country to the National Reference Laboratory for confirmation per national regulations. Human isolates included in the study originated from incident cases suspected of occupational exposure in dairy farms and from cases related in time and space but without any known exposure to dairy farms. The latter represents the broader epidemiological context of human brucellosis in Israel, particularly in the Negev region, which is known to be associated with exposure to small ruminants.

### Data acquisition

The following metadata were collected from laboratory records: date of sampling, indication for sampling, sample source material, biovar (routinely determined by the Reference Laboratory), name and location of the farm, and farm size (small [<300 cows], medium [300–1,000] or large [>1,000]).

Cases and corresponding samples were grouped into epidemiological clusters based on spatio-temporal considerations, including date of incidence, established commercial or geographic connection, or known movement of cattle between farms.

### Sample processing

DNA was extracted from 112 *B. melitensis* isolates by heat killing (80 °C, ten minutes), then extracted using the DNeasy Blood and Tissue kit (QIAGEN, Hilden, Germany). Genomic libraries were prepared with the Nextera Flex kit (Illumina, San Diego, CA, USA) and subject to paired-end sequencing using Illumina Miseq or Nextseq platforms.

### Sequence analysis

All 112 unique sequences were subject to quality control, trimming, and *de novo* assembly (Table S2) using the INNUca pipeline https://github.com/B-UMMI/INNUca (accessed in March 2022) with Trimmomatic (v0.36) [19], SPADes (v3.12.0) [20] and Pilon (v1.23) [21]. An additional 118 Israeli *B. melitensis* genome assemblies [17] were downloaded from the European Nucleotide Archive (bioproject PRJEB48426). All assembled genomes were checked for quality using QUAST (V.5.0.2) [22] and were *in silico* sequence-typed using the tool mlst (v2.17.6) https://github.com/tseemann/mlst (accessed in February 2022) with the *Brucella* spp. 9-loci scheme from pubMLST [12, 23, 24] (Table S3).

### cgMLST analysis

Two separate ad hoc cgMLST schemas were generated using chewBBACA (v2.6.0) [25] (with a Prodigal [26] training file for the reference genome *B. melitensis* 16M, and including loci with 95 % genome presence), including: [1] 92 bovine and bovine-related cohort isolates selected from the 112 unique isolates included in this study (2874 loci) and [2] 192 isolates from Southern Israel only, selected from the total of 230 available genomes, including bovine and bovine-related isolates together with bovine unrelated human isolates (2918 loci) (Tables S2 and S3). Minimum spanning trees (MSTs) were generated (with MSTreeV2) and visualised from each of the two ad hoc cgMLST schemas using GrapeTree [27]. Nodes were colour-coded according to the year of isolation, culture source, biovar, or assigned epidemiological clusters.

Isolates exhibiting fewer than ten differing alleles were considered as genomically related, and those exhibiting 10–15 differing alleles were considered possibly related. These thresholds were derived based on the studies of Janowicz *et al.* and Pelerito *et al.* [28, 13] and were further modified per our experience with genomic typing of the organism in Southern Israel [17, 29].

### SNP analysis

The phylogenetic analysis was also carried out using SNPs and depicted as MSTs but also using rooted trees to explore phylogenetic ancestry.

Complete genome assemblies of *B. melitensis* were downloaded from the NCBI RefSeq database using the datasets tool [30] (v.13.24.3; using the command: datasets download genome --assembly-level complete_genome --include-gbff --assembly-source refseq taxon ‘Brucella melitensis’). A neighbour-joining tree was produced using Mashtree (v.1.2.0) with the 112 genome assemblies and 70 reference genomes from RefSeq, and the reference genome was selected along with other RefSeq genomes (Fig. S1).

The RefSeq genome GCF_002191455.1 was used as reference for read mapping using Snippy and Snippy-core [31] (v.4.6.0). Recombination events were masked from the resulting core genome SNPs (cgSNPs) alignment using Gubbins [32] (v.2.4.1; default parameters) and SNP-sites (v.2.5.1). GrapeTree [27] (v.1.5.0) was used to generate a minimum spanning tree (MST) from the cgSNPs alignment. A maximum likelihood (ML) phylogeny was also generated with 1000 bootstraps using IQ-Tree2 [33] (v.2.1.2; with automated model selection using ModelFinder [34], the K3Pu+F+ASC+R2 model was used). The resulting bootstrapped ML tree was rooted on the most divergent reference genome in the analysed dataset -GCF_003856415.1). Figures with overlayed metadata were generated with the R packages ape [17, 35–37] (v.5.7), ggtree (v.3.4.2) and ggplot2 (v.3.4.1).

### Pangenome analysis

The 193 *B. melitensis* genome assemblies from southern Israel (this study and previous studies [17]) were annotated with prokka [38] (v.1.14.6), and the resulting GFF annotation files were used as input to panaroo [39] (v.1.3.2; with the parameter ‘--clean-mode strict’). The pangenome consisted of 3111 core genes (present in >99 % genomes), 10 soft core genes (present in between 95–99% genomes), 16 shell genes (present in between 15–95 %), and 34 cloud genes (present in between >0 to 15 % genomes). The resulting presence/absence matrix was then used to test for statistically significant gene differences between bovine and non-bovine isolates using scoary (v.1.6.16; using the Benjamini-Hochberg adjustment ‘-c BH’ and 1000 permutations ‘-e 1000’).

### Data analysis

The primary analysis (*n*=92) included only cases of brucellosis affecting dairy farms and secondary cases representing human exposure to infected cattle. MSTs were visually inspected to identify genomic clusters. These clusters were further investigated for epidemiological relatedness, based on available data, to confirm or refute suspected clusters and identify possible unrecognised clusters. Clusters were checked for geographic distances between the farms and for geographic clustering.

The secondary analysis (192 isolates) included the subset of isolates from the bovine-related cohort originating from Southern Israel (*n*=66) and 126 human cases of brucellosis occurring between the years 2014–2019 in Southern Israel. Of 126 human cases, 116 isolates were from the years 2017–2019 (recent publication, Zilberman *et al*. [17]), and ten were from the years 2014–2017 (included among the 112 unique isolates described above). These isolates were chosen since most bovine cases and clusters included in this study originated from that region. These cases had no known exposure to implicated dairy farms and represent brucellosis endemicity in the region, thus providing a broader epidemiological context for this organism.

### Statistical analysis

The χ^2^ test was used for categorical data as appropriate, using SPSS version 25 (IBM Corp Armonk, NY, USA). A two-tailed *P* value of ≤0.05 was considered significant.

### Data availability

The data for this study have been deposited in ENA at EMBL-EBI under accession number PRJEB52526 (https://www.ebi.ac.uk/ena/browser/view/PRJEB52526) and made public. Accession numbers of the individual samples are listed in Table S5 in the supplementary material. The data were also submitted to PubMLST.

## Results

**Fig. 1.**
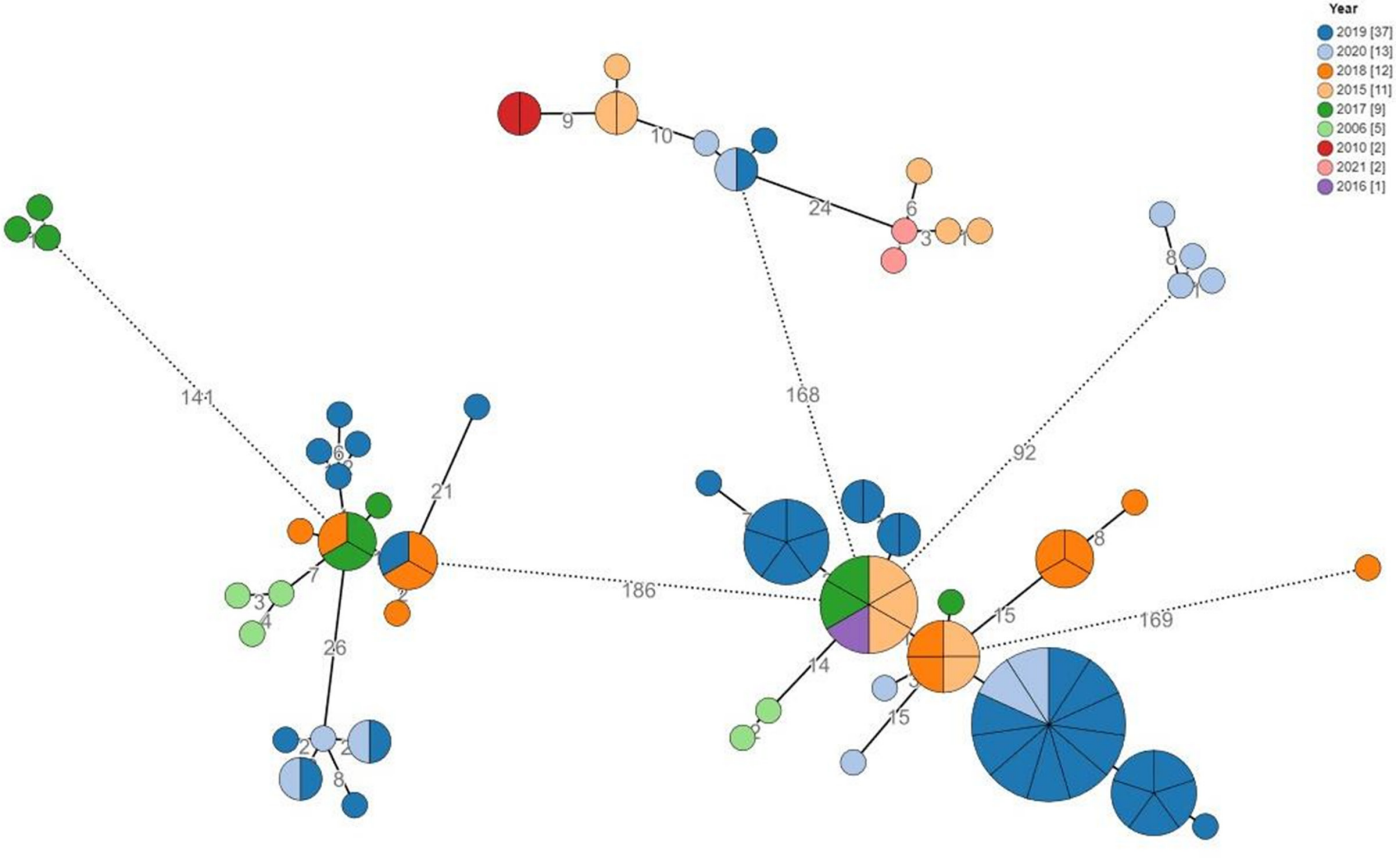
cgMLST analysis by year of isolation. Phylogenetic analysis of the bovine-human isolate cohort (*n*=92) based on an ad hoc cgMLST scheme of 2 874 alleles. Colour coding denotes year of sample acquisition. Node size is proportional to the number of isolates assigned to clone types. Numbers denote the allelic distances between nodes.

**Fig. 2.**
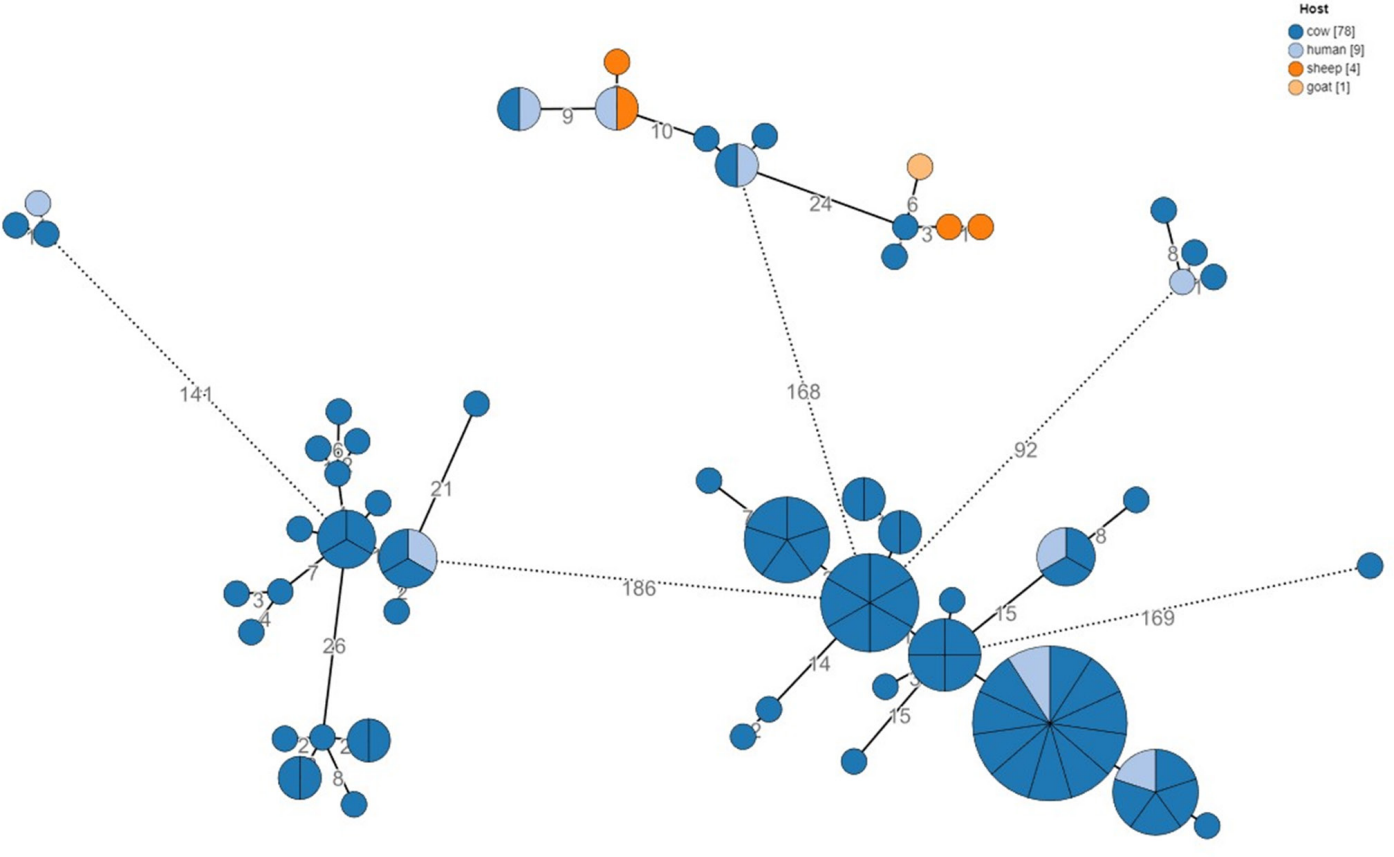
cgMLST analysis by source of isolation. Phylogenetic analysis of the bovine-human isolate cohort (*n*=92) based on an ad hoc cgMLST scheme of 2 874 alleles. Colour coding denotes the source of isolation. Node size is proportional to the number of isolates assigned to clone types. Numbers denote the allelic distances between nodes.

Study isolates included in the primary analysis were from 2006 to 2021, although the majority (85/92, 92.4 %) were from 2015 onwards. The isolates were grouped into 18 epidemiological clusters derived from 23 investigated incidents in dairy farms and animal husbandries in the time period specified. These 18 clusters include cattle from 19 different dairy farms hosting 8161 cows (farm characteristics are presented in Table S4). A total of 92 bovine-related isolates were sequenced and included in the analysis (Table 1); 78 were bovine isolates, five isolates originated from small ruminants (four sheep and one goat) and nine were epidemiologically related clinical human isolates. All human cases included were sourced from positive blood cultures. Of the bovine samples, 75 were obtained from milk and three from abortion material (Table 1). Geographically, 66 of 92 samples (71.7 %) originated from the southern region, and 23 of 92 samples (25 %) originated from northern Israel. The cgMLST trees of these 92 isolates are shown with designation of isolation year and by source of isolates in (Figs 1 and 2), respectively.

**Table 1.** Basic epidemiological characteristics of included dairy farms sorted by epi-cluster

Epi-cluster	Genomic cluster	Isolates by year									Isolates by source			Total
		2006	2010	2015	2016	2017	2018	2019	2020	2021	**Bovine**	**Ovine**	**Human**	
2	C7			2								2		2
4	C1	5	2	5	1	3	2		2		19		1	20
5	C6			2								1	1	2
9	C9					3					2		1	3
10	–						1				1			1
11	C4						4				3		1	4
12	C1							15	1		14		2	16
14	C2					1	3	1			4		1	5
15	C2					2	2	2			6			6
16	C5							2	2		3		1	4
18	C3							4	2		6			6
19	C1							4			4			4
20	C1							5			5			5
21	C2							4			4			4
22	C8								6		5		1	6
23	C7									2	2			2
24	C6			1								1		1
28	C7			1								1		1
Grand Total		5	1	5	1	8	10	35	11	2	78	5	9	92

### Biovar distribution

**Fig. 3.**
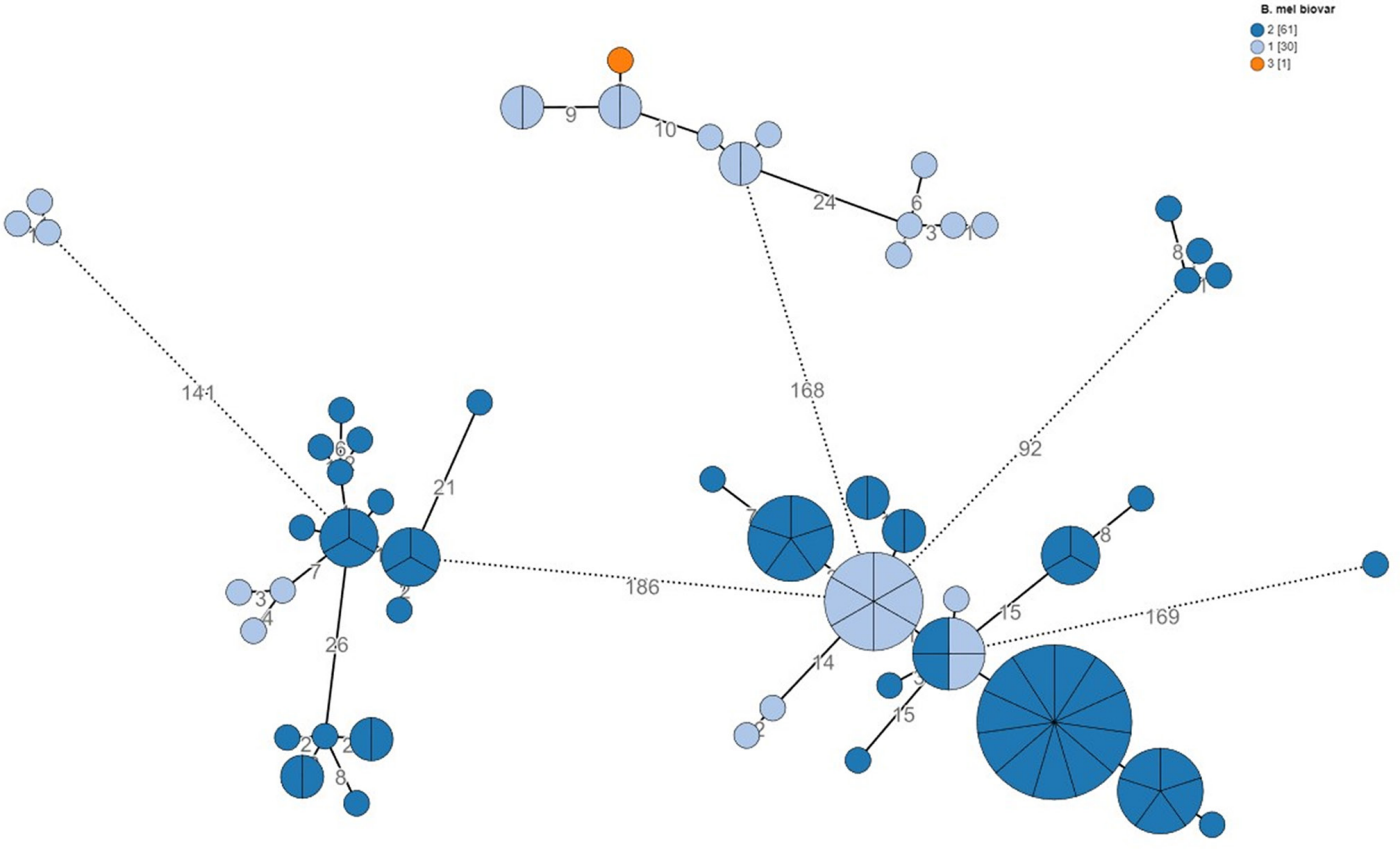
cgMLST analysis by biovar. Phylogenetic analysis of the bovine human-isolate cohort (*n*=92) based on an ad hoc cgMLST scheme of 2 874 alleles. Colour coding denotes the biovar of isolates. Node size is proportional to the number of isolates assigned to clone types. Numbers denote the allelic distances between nodes.

Of analysed isolates, 61 (66.3 %) belonged to *B. melitensis* biovar 2 and 30 (32.6 %) were biovar 1. Only one isolate (1.1 %) belonged to biovar 3. Isolates recovered before 2018 (*n*=28), were predominantly biovar 1 (24/28, 85.7 %), while among later isolates recovered from 2018 and onwards (*n*=64), only 6/64 (9.4 %) belonged to biovar 1 and 58/64 (90.6 %) belonged to biovar 2 (*P* for difference <0.001). The distribution of biovars across phylogeny is presented in Fig. 3.

### Genomic epidemiology

MLST analysis of the samples shows that most isolates belonged to sequence type (ST) 8 (Table S3), reconfirming that the ‘classical’ 9-MLST alone does not have sufficient discriminatory power for local brucellosis investigations.

**Fig. 4.**
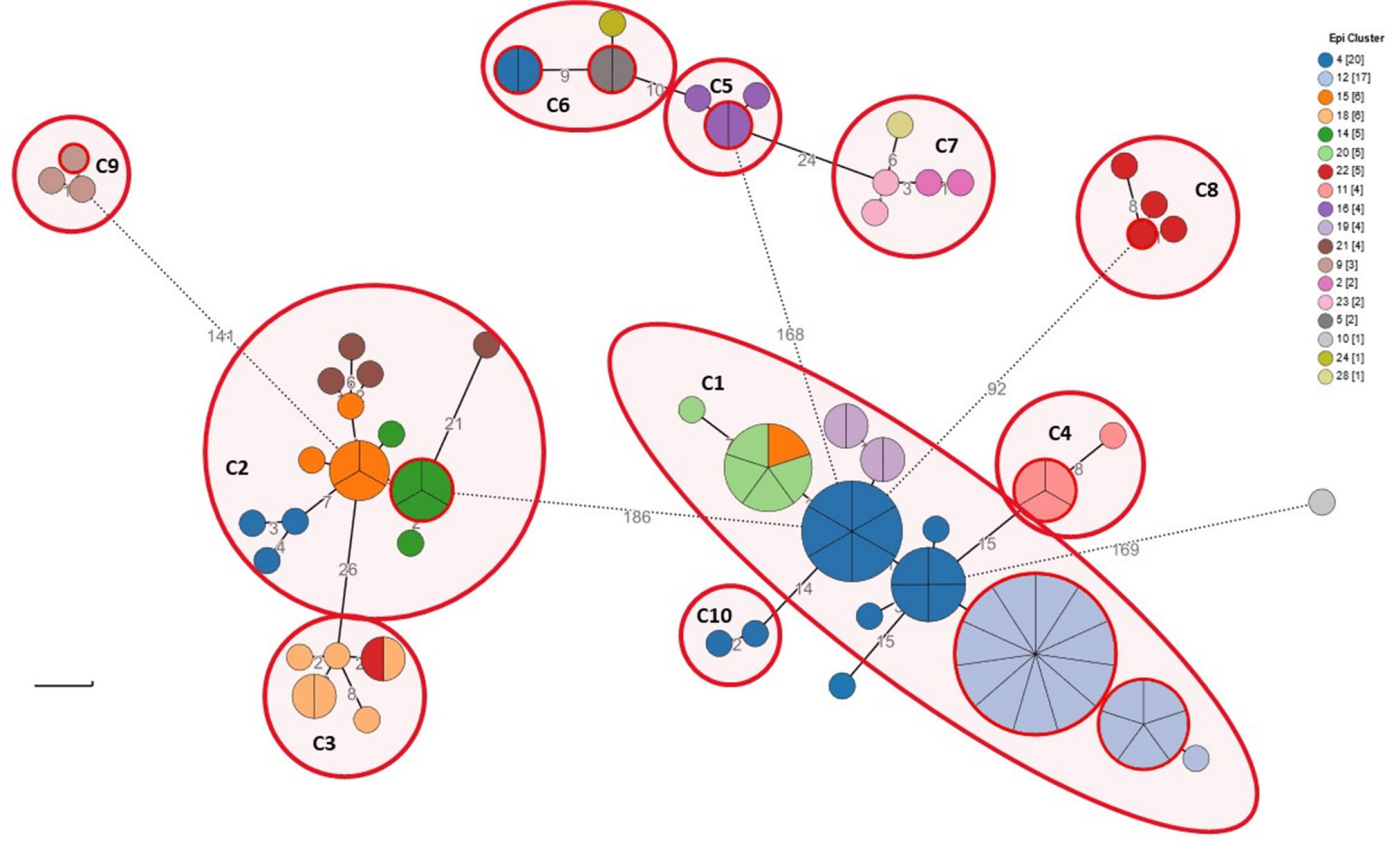
cgMLST analysis of epidemiological and genomic clusters. Phylogenetic analysis of the bovine-human isolate cohort (*n*=92) based on an ad hoc cgMLST scheme of 2 874 alleles. Colour coding denotes the investigated epidemiological clusters. Outer red rings on nodes represent human infections. Node size is proportional to the number of isolates assigned to clone types. Numbers denote the allelic distances between nodes. Outlines define genomically related isolates (genomic clusters).

The isolates were grouped into ten genomic clusters by inspecting the MST for allelic distances (Fig. 4) and compared with the different epidemiological clusters. The clusters were labelled C1-C10 as highlighted in Fig. 4. The phylogenetic tree demonstrates three large clades: genomic clusters C1-C4-C10, clusters C2-C3 and clusters C5-C6-C7. Generally, isolates within clusters were geographically related. However, the three major clades contained samples from different localities. Notably, C8 and C9 and a singleton isolate (B69, belonging to epi-cluster 10) are distinct from these clades.

Genomic clusters from northern Israel – C3, C7, C8 as well as the discrete cluster C9, are congruent with the initial epidemiological clustering and do not appear to be interconnected genomically with each other. The C4 cluster, while originating from the northern area, appears possibly related to the C1 cluster (15 differing alleles) and the parent clade.

**Fig. 5.**
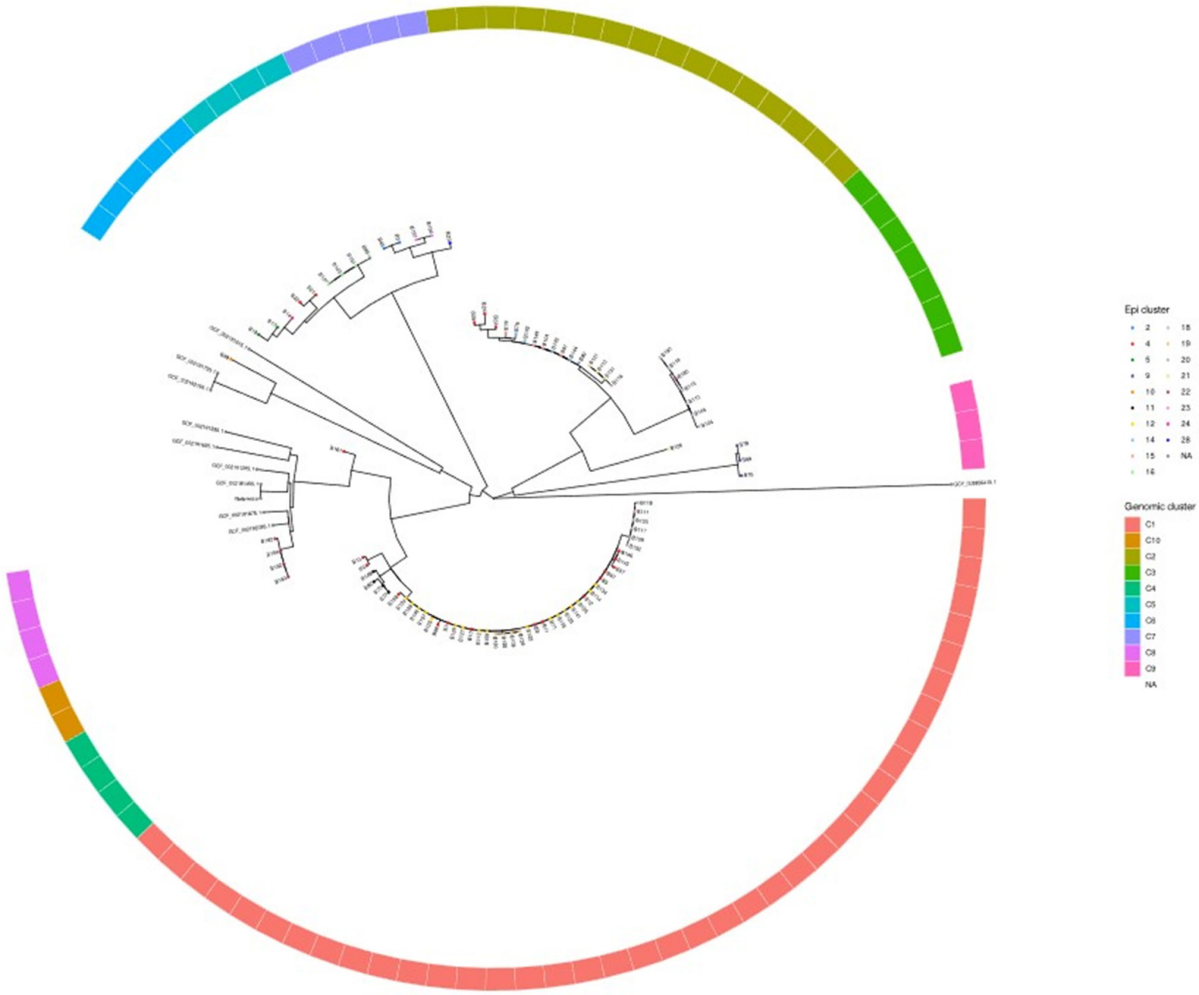
A rooted SNP-based analysis of bovine cohort. A rooted SNP tree of samples included in the human-bovine cohort (*n*=92). The reference genomes were selected as shown in Fig. S1. Leaf colours denote epidemiological cluster of the sample while the outer circle denotes the genomic cluster as defined in the text and in Fig. 4).

The above analysis was complemented by a core genome SNP tree (Fig. S2) which demonstrated a genomic clustering that agrees with the cgMLST analysis. When examining the rooted SNP-based tree (Fig. 5), the distinct cluster C8 appears to have diverged from the C1-C4-C10 clade and cluster C9 appears to have a common ancestral origin as the C2-C3 clade.

### Detailed genomic cluster analysis

The C1 cluster was the largest, notably harbouring isolates derived from four distinct epidemiological clusters with no apparent epidemiological connection. Isolates from different farms exhibited clonal relatedness within the cluster, confirming interconnections between the outbreaks. All C1 isolates were retrieved between 2016–2020. The farms involved in the C1 cluster (farms 1, 10, 15, 16) share a geographic area in the northern Negev region. As indicated by the rooted tree, epi-clusters 4 and 12 appear intermixed within C1, while epi-clusters 15 and 20 appear to have diverged from them. Cluster C10, which originated from farm 1 in 2006, was classified as possibly related based on allelic distance on the MST (Fig. 4), but its position within the clade and the epidemiological connection between genomic clusters suggested prolonged circulation of that strain in the region. Indeed, the rooted tree suggests C10 is part of the clade having a common ancestor for clusters C4 and C10.

Three farms [11, 12, 17] located in the same agricultural community are included in cluster C2. That cluster also contains three isolates (coloured blue, epi-cluster 4, Fig. 4) that were initially considered part of the outbreak in the farms belonging to cluster C1 during the epidemiological investigation. These isolates are genomically related and appear to have originated from three farms in a different community in southern Israel (farms 4, 5, 6), all recovered in 2006. The rest of the isolates in the cluster (epi-clusters 14, 16, 21) were collected between 2017 and 2019.

Cluster C6 includes five genetically related isolates, including one bovine isolate, two human isolates, and two ovine isolates. Two identical isolates include a bovine isolate and a clinical isolate from a human case – an employee at the same farm (farm 3). An additional pair of identical isolates includes an ovine isolate and a clinical isolate from an epidemiologically related human case. The other singleton ovine case is from a town in the southern region relatively close to the farm (19 km). The isolates implicated in ovine-human transmission (epi-cluster 5) were isolated in a large Arab city in northern Israel and according to the rooted SNP tree appear to have occurred later than the single ovine case, thus implicating a plausible direction of transmission (ovine-to-ovine-to-human). The bovine-human pair appears to have a common ancestor with this ovine sub-cluster. Cluster C6 is genomically related to cluster C5 from a farm in southern Israel, 37 km away from the southern city and 21 km from the farm in cluster C6.

Secondary human cases related to dairy farm exposure were found in clusters C1, C2, C4, C5, C6, C8, and C9 and are elaborated in Table 1. These human cases were identified as having a direct connection to bovine cases through epidemiological investigation and epi-cluster identification. These cases, affecting farm owners or animal handlers, each involve a clone infecting both cattle and human subjects and reaffirm the epidemiological data. The rooted SNP analysis does not show clear directionality between human cases and their source, likely reflecting that human and animal isolates were commonly sampled simultaneously during investigation.

### Secondary genomic analysis

**Fig. 6.**
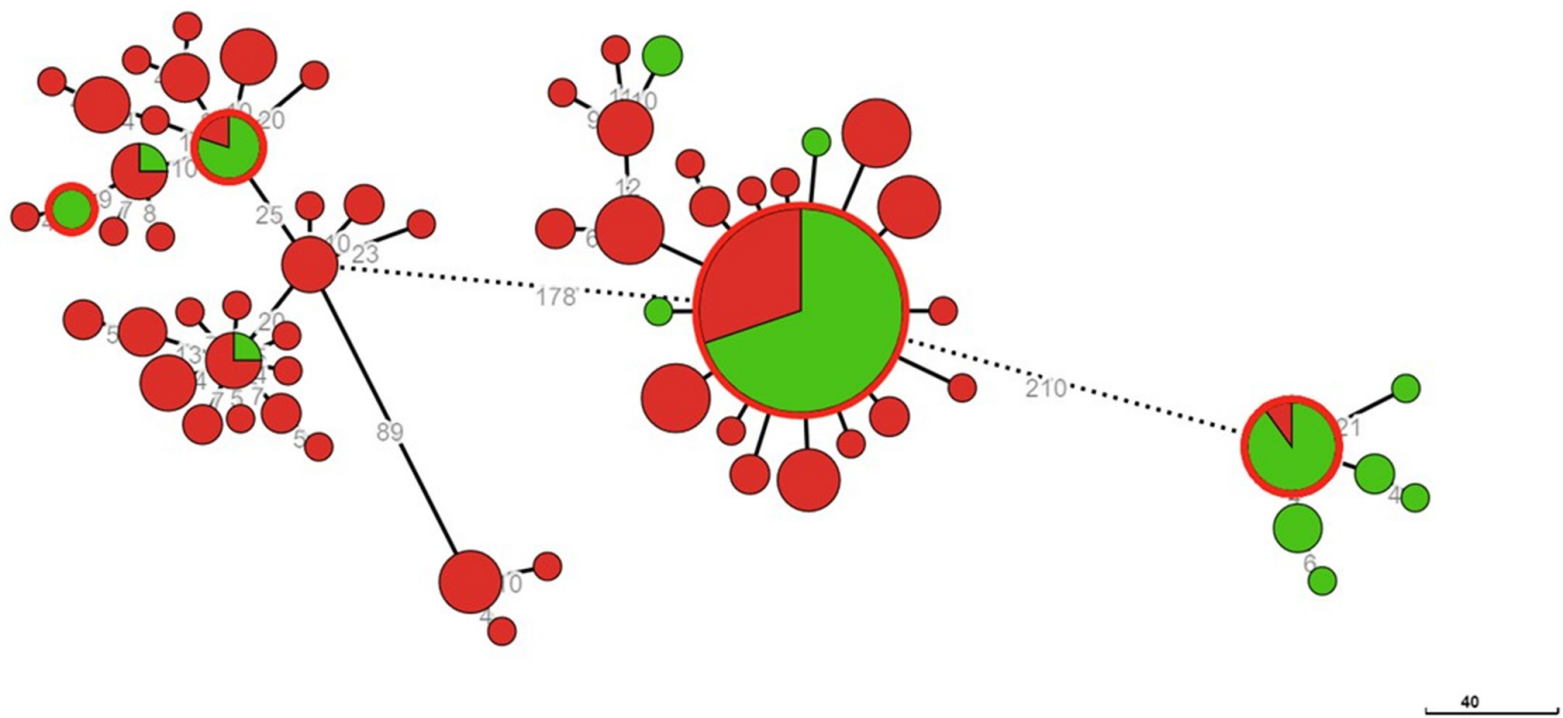
Secondary cgMLST analysis of bovine and non-bovine related cohorts. Combined phylogenetic analysis of the isolates originating from Southern Israel in the bovine-human isolate cohort (green, *n*=66) and clinical-human cohort (red, *n*=126) based on an ad hoc cgMLST scheme of 2 918 alleles. Node size is proportional to the number of isolates assigned to clone types. Numbers denote the allelic distances between nodes. Branches shorter than three alleles were truncated. Outer red rings denote human isolates from the bovine-human cohort.

The secondary analysis included 192 isolates from southern Israel only (126 representing endemic human brucellosis and 66 representing bovine and bovine-related isolates). Fig. 6 shows the combined MST tree for this analysis. Three clades are readily noticeable in this tree. The right-sided clade contains mainly bovine and bovine-related samples, the left clade contains mostly human clinical samples, while the middle clade is intermixed between the two subgroups. The clade to the right side contains only one human isolate and a tightly grouped cluster from the bovine cohort. This clinical sample originated from the exact location as the farms in this cluster (farms 11, 12, 21 – all located in the same agricultural community in southern Israel – corresponding to cluster C2), and thus represents a bovine outbreak with a secondary human case.

The remaining isolates are intermixed with a few evident clusters. No apparent clustering is seen according to the community of residence or by the ethnic classification of the community. This clustering pattern represents many sporadic human cases within Bedouin communities in Southern Israel that are likely associated with unpasteurised ovine and caprine milk consumption and occasional local transmission to dairy cattle.

**Fig. 7.**
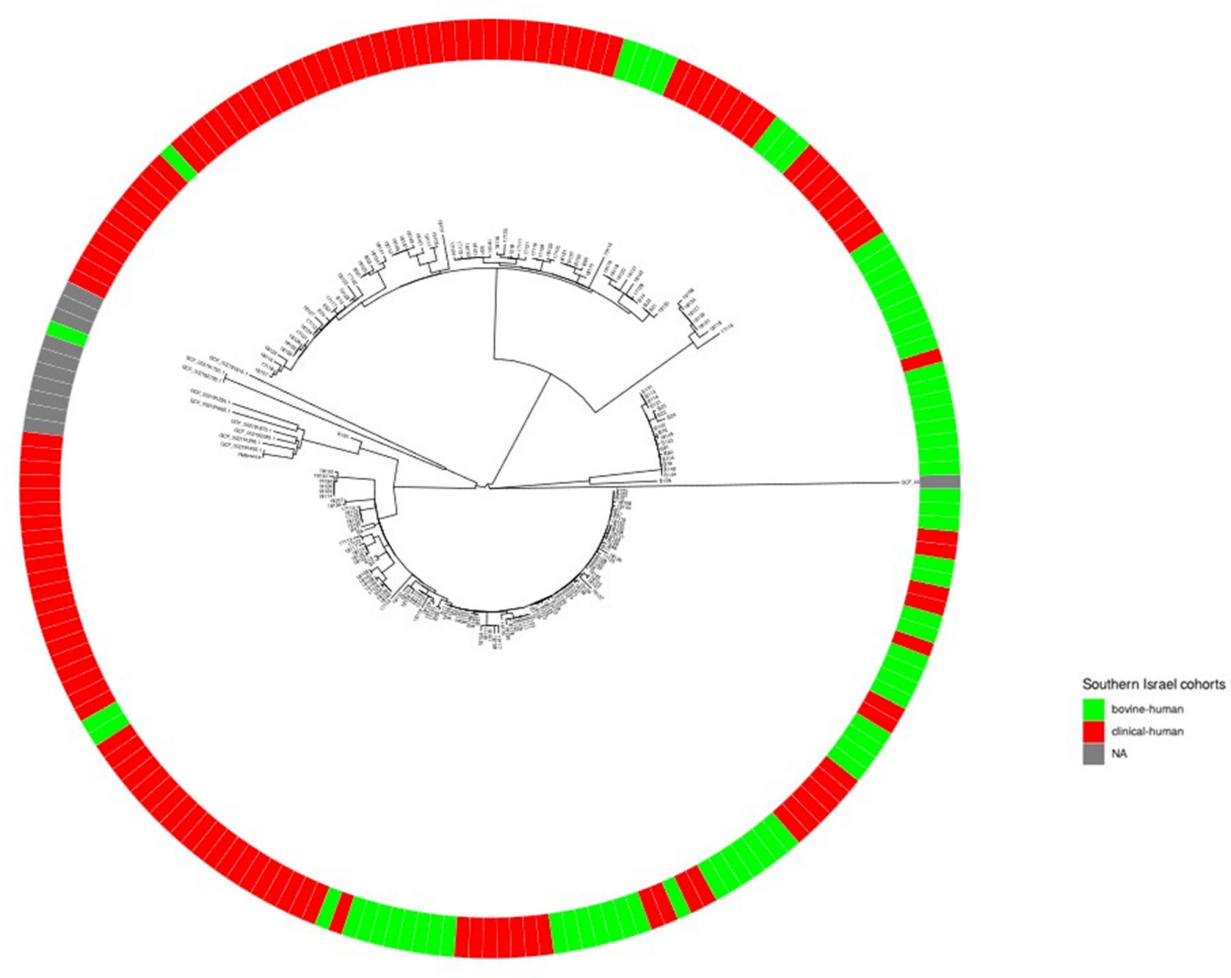
A rooted SNP-based secondary analysis. A combined rooted SNP tree of samples originating from Southern Israel belonging to the bovine-human cohort (green, *n*=60) and the clinical human cohort (red, *n*=126).

A SNP analysis of the secondary dataset is depicted on a rooted tree in Fig. 7. Notably the tree demonstrates that the bovine isolates are intermixed with the endemic isolates unrelated to dairy farms, including cases where bovine isolates appears to have diverged from endemic cases and vice versa.

### Pan-genome analysis

An initial pan-genome comparison carried out by comparing the bovine isolates in our cohort to non-bovine isolates in the cohort, found no significant differences in the presence or absence of genes between the two groups (data not shown). A further comparison was carried out among isolates from Southern Israel (*n*=193), of which 60 were of bovine origin and the remainder were non-bovine (Table S6). The analysis identified several statistically significant differences in gene presence / absence, involving certain transposases or hypothetical proteins, all of which were identified among the two groups but at varying percentages.

## Discussion

Our study is the first to demonstrate the sustained dissemination of *B. melitensis* into dairy farms in an endemic country. By applying WGS to a large cohort of veterinary and clinical isolates, we were able to identify transmission chains traversing One Health in the region, affecting small ruminants, dairy cows, and humans. Our findings suggest that bovine infections in dairy farms represent a spillover from small ruminants, the natural reservoir of *B. melitensis* in the region. Like the widespread endemic human infection in Southern Israel, secondary to continued exposure to infected sheep and goats, the dissemination of brucellosis in dairy farms resulted in secondary human infections.

In addition to human disease, *B. melitensis* has grave implications for cow welfare and milk production and is associated with immense control efforts [5, 40] within farms, such as test-and-slaughter practices.

### Genomic epidemiology as an essential tool in brucellosis outbreak investigations

The connections between implicated dairy farms identified using traditional and genomic epidemiology show notable congruence. In our phylogenetic analysis, cases deemed closely related according to the epidemiological investigation commonly cluster within the same genomic clusters (Fig. 4). The genomic data further refined the epidemiological picture in areas of uncertainty. For example, a case of brucellosis in a veterinarian working in dairy farms in southern Israel (B103, Table S1), epidemiologically related to two of the farms affected by brucellosis outbreaks (farms 10 and 13). The concurrent occupational exposure to both farms rendered the source of infection unidentified. However, WGS confirmed farm 13 as the source of infection in this case.

Genomic cluster C1 is linked to outbreaks from several different farms – 1, 10, 15, and 16, all located in the northern part of the Negev region and farm 2 which is in the remote Arava region (South). While the former farms are in the same region, the aerial distances between farm 1 and farms 10, 15 and 16 are 30, 47 and 21 kilometres (km), respectively. Furthermore, no known movement of cattle, feeding material, or personnel was documented between these farms, except for a single sale of a young female cow from farm 1 to farm 2, which tested positive after being quarantined in farm 2 (isolate B7). While these farms share several spatio-temporal features and are confirmed to be related based on WGS analysis, traditional epidemiology could not confirm a link between infections in these farms.

Fig. 3 shows the biovar distribution in the investigated bovine outbreaks and its relation to phylogeny. *B. melitensis* biovars have traditionally been used to establish possible connections between cases when uncertainty about origin arises or to strengthen an existing connection. Our data show that closely related isolates exhibited different biovars, thus rendering the biovar classification non-specific. Moreover, the ‘classic’ MLST analysis places most of the isolates of our cohort in ST8. These limitations of traditional typing methods for brucellosis investigation have been shown previously and argue in favour of routinely applying WGS, as it has a far greater discriminatory power [13].

### Elucidating the reservoir and transmission between farms

Our data support a common, geographically dependent reservoir that connects the farms in an area, as seen in the Negev region. Such reservoirs and vehicles of transmission are likely local herds of sheep and goats, which harbour *B. melitensis* and transmit it to farm animals. Herds in the Negev area are relatively poorly regulated [41]. This involves both a low uptake of the Rev-1 vaccine and inadequate surveillance of at-risk animals. In cluster 6, a sheep isolate from a large Negev city was found related to a dairy farm isolate in the same region (a distance of 19 km). This provides an example of the interconnectedness of dairy farms and local herds of small ruminants in the same region. The same cluster also resulted in spillover from the dairy farm to humans, in that case, infection of an employee on the same farm. This relatedness is also evident from the SNP analysis.

As described above, cluster C2 consists of related isolates from 2017 to 2019 and 2006. The small sub-cluster of three isolates dating back to 2006, could represent the historical point of introduction of *B. melitensis* to the agricultural community. The detection of the genomic cluster to which the 2006 clone belongs during 2017–2019 in the same area, suggest a sustained circulation of this clone for many years and coincides with the slow mutation rate seen in *B. melitensis,* which enables genomic connections to persist over time [42].

### A clear connection between bovine and human brucellosis

In our cohort, a direct epidemiological link connects nine human cases to bovine cases (Fig. 2). All represent clear occupational exposures (farm owners, animal handlers and veterinarians). The phylogenetic analysis of bovine and bovine-related cases shows inter-mixing with clinical human isolates from that geographic area (southern Israel), including endemic cases having no links to dairy farms (which are presumably related to consumption of unpasteurised small ruminant milk) (Fig. 6). This finding coincides with the hypothesis that bovine brucellosis in the region represents another example of spillover of *B. melitensis* from the natural ovine reservoir. Further supporting this hypothesis is that most intermixed cases were no more than 20 alleles apart across the three cohorts, suggesting an ancestral genomic link between cases in that locality. The rooted SNP tree (Fig. 7) further supports this.

The spillover of *B. melitensis* from its natural host (sheep and goats) to dairy herds also raises the question of host adaptation of the pathogen in our region to bovine hosts. While comparing bovine and non-bovine isolates by means of a pan-genome analysis, several significant differences in gene presence / absence were found. However, these were either transposases or hypothetical proteins found at varying percentages among the two groups, and thus this finding has questionable biological significance. Whether bovines are accidental hosts of *B. melitensis* representing coincidental transmission opportunities from the natural reservoir or that certain strains might have a predilection towards infecting bovine hosts, deserves further study.

### Defining clusters and genomic relatedness

In the 2018 work by Janowicz [13, 28], an allelic distance of seven alleles or a difference in seven SNPs was considered to be the threshold for determining relatedness between isolates. However, this study was not designed to specifically address this question. Also, the dataset included in that study might have not been ideal for the purpose of validating relatedness thresholds as many included isolates were tightly clustered or notably distant. Interestingly, Pelerito *et al*. in 2020 [28], addressed this specific question and proposed an allelic distance of 0.4 % (roughly equivalent to 10–11 differing alleles) to best represent epidemiologically-clustered isolates. Our own work, describing an outbreak investigation [7] of *B. melitensis* in Israel, originating from contaminated camel milk, clearly related isolates that had a SNP distance of up to 13. Therefore, we believe that a distance up to 10 alleles or 13 SNPs, was well-suited to infer relatedness in our cohort. That said, establishing globally applicable thresholds should be subject to constant refining as additional studies exploring the genomic epidemiology of this pathogen will accumulate.

### Study limitations

This study has several limitations. First, this is a retrospective study, using historical isolates from the national repository, recovered through sampling performed during outbreak investigation over many years. Therefore, our dataset does not represent a systematic surveillance of dairy farms. Second, we were able to include only a small number of isolates recovered from small ruminants, which hinders our ability to establish the role of local ovine herds as vehicles of transmission between farms. This limitation stems from the fact that the dairy industry is tightly regulated and thus any suspected animal or related human infection is subject to microbiological investigation, while small ruminants are not subject to a similar level of inspection, rendering the isolation of *B. melitensis* from these animals rare. This limitation hinders our ability to consistently establish ovine-bovine genomic links, and particularly to infer the directionality of transmission. We tried to overcome these limitations and enhance the understanding of the local epidemiology by including the secondary analysis of human clinical isolates from the same geographic area, which are known to become infected by sheep and goats in our region, and point to a common, geographically dependent source.

## Conclusions

This study shows a persistent and widespread circulation of *B. melitensis* in dairy farms in Israel. The genomic epidemiology uncovers previously cryptic connections between outbreaks in different farms that share a general geographic area, with no other apparent connections. We also show the widespread regional connection between bovine and human brucellosis; these infections likely share a common reservoir that results in endemic transmission, but occupational transmission also occurs in dairy farms. We hypothesize that this common source is the local small ruminant herds in the region, however, the small number of ovine isolates included limits our ability to confirm this.

We believe our data shows that controlling transmission to and between dairy farms and humans is inseparable and that this public health challenge must be addressed using a One Health approach.

While bovine brucellosis is mainly attributed to *B. abortus *, we show that *B. melitensis* can potentially become a significant pathogen affecting bovine health following the elimination of *B. abortus *. Infections in dairy farm cows carry severe economic implications and affect animal health and welfare; they also serve as a source for onward human infection. Further research should focus on the risk factors and transmission dynamics of *B. melitensis* between small ruminants and dairy herds. Since *B. melitensis* is yet uncontrolled in many parts of the world, public health and veterinary risk assessments should consider its potential spread beyond ovine and caprine herds. Management of this risk necessitates appropriate epidemiological and microbiological surveillance and implementation of control measures such as veterinary inspection and vaccination across the entire range of farm animals likely to become affected by this neglected zoonosis.

## Supplementary Data

Supplementary material 1
