# Supplementary material for: Public and animal health risks associated with spillover of Brucella melitensis into dairy farms
Source: Microb Genom. 2023 Apr 28;9(4):mgen001014. doi: 10.1099/mgen.0.001014 (PMC10210956; doi:10.1099/mgen.0.001014)

Supplementary material - Public and Animal Health Risks Associated with Spillover of *Brucella melitensis* into Dairy Farms

Table of contents:

Page	
2	Table S1 - Details of samples included in primary analysis
6	Table S2 - Raw sequence stats and assembly stats for 238 Israeli *B. melitensis* isolates.
14	Table S3 - In silico sequence typing results for 238 Israeli *B. melitensis* isolates.
22	Table S4 – General farm information
24	Table S5 – Accession numbers for sequenced isolates
28	Figure S1 – Reference genome selection
30	Table S6 – Results of pan-genomic analysis
31	Figure S2 - Phylogenetic analysis of the bovine-human isolate cohort (n=92) based on an ad hoc cgSNP scheme

Table S1. Details of samples included in primary analysis (n=92)

Sample ID	Epidemiological cluster	Farm code	Year	Geographic area	Host species	Material	*B. melitensis* biovar	accession number
B 10	4	1	2015	South	cow	Milk	1	243519
B 103	16	13	2019	South	human	Blood	1	350545
B 104	15	12	2018	South	cow	Milk	2	E224457
B 106	18	14	2020	North	cow	Milk	2	375052
B 107	16	13	2020	South	cow	Milk	1	375701
B 108	19	15	2019	South	cow	Milk	2	356155
B 109	20	16	2019	South	cow	Milk	2	E232712
B 11	4	1	2015	South	cow	Milk	1	243929
B 110	18	14	2019	North	cow	Milk	2	361968/7
B 111	15	12	2019	South	cow	Milk	2	E232307
B 112	12	10	2019	South	cow	Milk	2	E231901
B 113	21	17	2019	South	cow	Milk	2	E231715
B 114	12	10	2019	South	cow	Milk	2	E231765
B 115	18	14	2019	North	cow	Milk	2	232881/7
B 116	15	12	2019	South	cow	Milk	2	E232432
B 117	20	16	2019	South	cow	Milk	2	E232431/10
B 118	20	16	2019	South	cow	Milk	2	E232431/4
B 119	18	14	2019	North	cow	Milk	2	232881/1
B 12	4	1	2015	South	cow	Milk	1	243518
B 120	12	10	2019	South	cow	Milk	2	E231765
B 121	21	17	2019	South	cow	Milk	2	E231407
B 122	12	10	2019	South	cow	Milk	2	E233227
B 123	12	10	2019	South	cow	Milk	2	E231901

B 124	18	14	2019	North	cow	Abortion	2	361968/1
B 125	12	10	2019	South	cow	Milk	2	E230157
B 126	21	17	2019	South	cow	Milk	2	E233336
B 127	12	10	2019	South	cow	Milk	2	E232099
B 128	12	10	2019	South	cow	Milk	2	E232173
B 129	12	10	2019	South	cow	Milk	2	E232099
B 13	4	1	2006	South	cow	Milk	1	537176
B 130	19	15	2019	South	cow	Milk	2	E229717
B 131	21	17	2019	South	cow	Milk	2	E230974
B 132	20	16	2019	South	cow	Abortion	2	E230359
B 133	20	16	2019	South	cow	Milk	2	E231284
B 134	12	10	2019	South	cow	Milk	2	E232173
B 135	12	10	2019	South	cow	Milk	2	E230558
B 137	12	10	2019	South	cow	Milk	2	E231589
B 138	19	15	2019	South	cow	Milk	2	356155
B 139	19	15	2019	South	cow	Milk	2	356155
B 14	24	20	2015	South	sheep	Milk	3	E20104
B 140	12	10	2019	South	cow	Milk	2	E232173
B 141	12	10	2019	South	cow	Milk	2	E232048
B 142	14	11	2019	South	cow	Milk	2	E231183
B 143	14	11	2018	South	cow	Milk	2	317807
B 144	14	11	2018	South	cow	Milk	2	223922
B 145	4	1	2018	South	cow	Milk	2	335346/1
B 145	11	9	2019	North	cow	Milk	2	335346
B 146	4	1	2018	South	cow	Milk	2	335346/5
B 146	11	9	2019	North	cow	Milk	2	335346
B 148	11	9	2018	North	cow	Milk	2	331917
B 149	15	12	2018	South	cow	Milk	2	E224430
B 150	18	14	2020	North	cow	Milk	2	385840
B 151	16	13	2020	South	cow	Milk	1	385867/3

B 152	22	18	2020	North	cow	Milk	2	395724
B 153	22	18	2020	North	cow	Milk	2	399140
B 154	22	18	2020	North	human	Blood	2	385419
B 155	12	10	2020	South	human	Blood	2	382356
B 156	23	19	2021	North	cow	Milk	1	E240050
B 157	23	19	2021	North	cow	Milk	1	E240050
B 158	4	1	2020	South	cow	Milk	2	E237042/2
B 159	12	10	2020	South	cow	Milk	2	387221
B 160	22	18	2020	North	cow	Milk	2	387416/1
B 161	4	1	2020	South	cow	Milk	2	E237042/1
B 162	22	18	2020	North	cow	Milk	2	386355
B 17	5	23	2015	North	sheep	Milk	1	221146/2
B 19	5	23	2015	North	human	Blood	1	241051
B 2	2	22	2015	North	sheep	Milk	1	E19989
B 20	28	21	2015	South	goat	Milk	1	228127
B 21	4	3	2010	South	human	Blood	1	72315
B 22	4	3	2010	South	cow	Milk	1	E3534
B 23	4	4	2006	South	cow	Milk	1	543937/410
B 24	4	5	2006	South	cow	Milk	1	539107
B 25	4	6	2006	South	cow	Milk	1	543084
B 5	4	1	2015	South	cow	Milk	1	E21268/3
B 57	4	1	2017	South	cow	Milk	1	220569/1
B 6	4	1	2006	South	cow	Milk	1	528119
B 65	2	22	2015	North	sheep	Milk	1	E19989
B 66	4	1	2017	South	cow	Milk	1	E220308
B 67	4	1	2017	South	cow	Milk	1	E220336
B 68	9	7	2017	South	cow	Milk	1	306852
B 69	10	8	2018	North	cow	Milk	2	E224523
B 7	4	2	2016	South	cow	Milk	1	E50501
B 70	11	9	2018	North	cow	Milk	2	E225638

B 71	12	10	2019	South	human	Blood	2	346511
B 75	9	7	2017	South	cow	Milk	1	303105/4
B 76	14	11	2018	South	human	Blood	2	319627
B 77	11	9	2018	North	human	Blood	2	323233
B 78	9	7	2017	South	human	Blood	1	312011
B 79	15	12	2017	South	cow	Milk	2	E224430
B 80	14	11	2017	South	cow	Milk	2	307706
B 81	15	12	2017	South	cow	Milk	2	E224457/1
B 82	11	9	2018	North	cow	Milk	2	E225641
B 85	16	13	2019	South	cow	Abortion	1	340007
B 9	4	1	2015	South	cow	Milk	1	242715

Table S2. Raw sequence statistics and assembly stats for 230 Israeli *B. melitensis* isolates.

		Reads		Assembly				
**ID**	**Reference**	**initial depth**	**downsampled depth**	**Number of contigs**	**GC content**	**Total assembly length**	**Average contig size**	**N50**
**B 10**	this study	121	121	49	54.67	3294299	67230.6	276432
**B 103**	this study	106.4	85.5	27	54.73	3290579	121873.3	276570
**B 104**	this study	100.2	82.2	26	54.82	3292708	126642.6	333741
**B 106**	this study	102.5	83.2	26	54.89	3291408	126592.6	299235
**B 107**	this study	87.1	70.6	24	54.31	3290064	137086	359117
**B 108**	this study	80.4	65.9	26	54.61	3290997	126576.8	296829
**B 109**	this study	69.2	56.1	24	54.53	3289669	137069.5	299223
**B 11**	this study	114	114	65	55.35	3485847	53628.4	383926
**B 110**	this study	66.9	54.7	29	54.68	3290228	113456.1	276786
**B 111**	this study	50	39.8	36	55.13	3292149	91448.6	171753
**B 112**	this study	56.8	45.6	30	54.43	3291129	109704.3	276463
**B 113**	this study	69	54.8	25	54.75	3292419	131696.8	291306
**B 114**	this study	106.7	85.6	26	54.25	3290241	126547.7	276464
**B 115**	this study	68.3	54.6	30	54.75	3291075	109702.5	296728
**B 116**	this study	70.8	57.5	30	55.03	3292605	109753.5	276552
**B 117**	this study	69.1	53.4	24	54.56	3290743	137114.3	299223
**B 118**	this study	53.7	42.1	676	56.43	3370854	4986.5	8171
**B 119**	this study	53	43.2	19	54.53	3292370	173282.6	531393
**B 12**	this study	106	106	78	54.66	3296909	42268.1	276439
**B 120**	this study	70.5	56.7	25	54.59	3291836	131673.4	276569
**B 121**	this study	52.8	41.3	457	56.23	3346127	7321.9	13057
**B 122**	this study	100	80.1	25	54.67	3291859	131674.4	299253
**B 123**	this study	78.3	62.4	27	54.97	3291490	121907	276466
**B 124**	this study	44.5	34.9	1911	56.76	3521050	1842.5	2699
**B 125**	this study	96.7	78.4	26	54.49	3289725	126527.9	276468
**B 126**	this study	78.2	62.6	282	56.83	3302015	11709.3	19193
**B 127**	this study	86.5	69	22	54.37	3291448	149611.3	333734
**B 128**	this study	107.2	86.5	20	54.56	3291068	164553.4	434655

**B 129**	this study	101.4	81.4	24	54.56	3291262	137135.9	299254
**B 13**	this study	99	99	78	54.79	3298020	42282.3	193123
**B 130**	this study	82	64.6	23	54.49	3292581	143155.7	358507
**B 131**	this study	73.9	58.3	27	54.52	3292038	121927.3	296811
**B 132**	this study	65.3	51	25	54.35	3290355	131614.2	299223
**B 133**	this study	95.9	76.9	25	54.47	3291388	131655.5	299253
**B 134**	this study	98	77.2	25	54.64	3291236	131649.4	299253
**B 135**	this study	80.8	64.9	28	54.44	3291229	117543.9	359060
**B 137**	this study	93	75.6	23	54.4	3291762	143120.1	358117
**B 138**	this study	60.7	47.8	26	54.75	3291381	126591.6	276507
**B 139**	this study	63.5	50.9	26	53.95	3290917	126573.7	299253
**B 14**	this study	111	111	84	54.58	3299383	39278.4	193132
**B 140**	this study	37.9	30.4	30	54.47	3291175	109705.8	249534
**B 141**	this study	75.9	61.7	21	54.65	3290710	156700.5	358506
**B 142**	this study	124.7	101	26	54.75	3291600	126600	299226
**B 143**	this study	78.6	64.3	25	55.22	3291153	131646.1	359044
**B 144**	this study	95.6	77.8	27	54.69	3291779	121917.7	291307
**B 145**	this study	61.2	48.4	26	54.89	3291400	126592.3	251061
**B 146**	this study	69.2	54.8	24	54.28	3291132	137130.5	276481
**B 148**	this study	44.4	34.7	1870	56.84	3513490	1878.9	2770
**B 149**	this study	104.4	83.6	28	55.07	3291939	117569.3	333766
**B 15**	this study	128	128	76	54.76	3296864	43379.8	359043
**B 150**	this study	86.2	69.3	29	54.86	3291610	113503.8	299152
**B 151**	this study	80.8	65.7	22	54.36	3290944	149588.4	359044
**B 152**	this study	67.7	54.8	26	54.48	3290043	126540.1	299348
**B 153**	this study	54.9	44.5	3094	57.23	3645332	1178.2	1618
**B 154**	this study	82.6	67.9	24	54.04	3291332	137138.8	359060
**B 155**	this study	86.7	67.5	19	54.52	3292132	173270.1	434662
**B 156**	this study	33.6	25.4	25	54.46	3290285	131611.4	251058
**B 157**	this study	46.2	36.6	24	54.56	3290242	137093.4	296803
**B 158**	this study	59.1	46.7	24	54.56	3291196	137133.2	299253
**B 159**	this study	71.2	56.7	25	54.78	3292773	131710.9	276466

**B 16**	this study	95	95	73	55.31	3296443	45156.8	249531
**B 160**	this study	75.6	60.9	26	54.57	3291974	126614.4	333868
**B 161**	this study	68	53.7	135	56.3	3296631	24419.5	52020
**B 162**	this study	68.7	55.2	26	54.52	3291150	126582.7	333730
**B 17**	this study	77	77	60	54.7	3296061	54934.4	276466
**B 18**	this study	133.7	106.8	33	55.01	3290654	99716.8	251042
**B 19**	this study	138.8	109.9	33	55.9	3290600	99715.2	251270
**B 2**	this study	148.2	123.4	36	55.29	3291177	91421.6	189612
**B 20**	this study	150	150	84	55.04	3371507	40137	193054
**B 21**	this study	47.1	40.5	27	54.37	3295970	122073	251042
**B 22**	this study	42.9	36.5	24	54.06	3297509	137396.2	365831
**B 23**	this study	52.3	45	31	54.53	3296928	106352.5	221764
**B 24**	this study	65	56.6	33	54.57	3297026	99909.9	293311
**B 25**	this study	50.5	43.7	32	54.83	3297131	103035.3	279308
**B 32**	this study	52.6	45.3	36	54.28	3297422	91595.10	221799
**B 35**	this study	53.6	46.1	31	54.75	3296955	106353.40	293267
**B 36**	this study	55.5	47.8	27	54.31	3296478	122091.80	293329
**B 37**	this study	60.2	52.3	31	54.31	3296704	106345.30	296829
**B 38**	this study	60.4	51.6	32	54.21	3297035	103032.30	251058
**B 39**	this study	61.2	51.5	88	56.01	3303612	37541	91739
**B 40**	this study	61	52.5	32	54.52	3296903	103028.20	293329
**B 47**	this study	102.7	77.1	27	54.45	3296311	122085.6	296821
**B 48**	this study	93.1	68.7	26	54.3	3296185	126776.30	359043
**B 5**	this study	140.2	112.4	35	55.04	3291147	94032.8	276432
**B 50**	this study	102.6	77.2	26	54.33	3296075	126772.10	359045
**B 51**	this study	115.5	86.9	24	54.15	3296069	137336.2	296832
**B 52**	this study	132	99.7	26	54.65	3296506	126788.7	296793
**B 53**	this study	106.4	79.1	27	54.32	3295934	122071.6	359045
**B 54**	this study	159.3	120	28	54.55	3296731	117740.40	296890
**B 55**	this study	155.2	116.7	29	54.5	3295827	113649.2	273820
**B 56**	this study	125.1	92.9	24	53.99	3296408	137350.30	519037
**B 57**	this study	80.4	74.5	27	54.21	3293735	121990.2	296729

**B 6**	this study	150.8	121	36	55.22	3291564	91432.3	193100
**B 65**	this study	116.9	88.1	26	54.63	3296226	126777.9	296802
**B 66**	this study	89.5	84	29	54.4	3293547	113570.6	293197
**B 67**	this study	82.1	76.6	30	54.31	3294245	109808.2	293197
**B 68**	this study	74.1	68.9	32	54.63	3294297	102946.8	276396
**B 69**	this study	86.3	80.4	28	54.4	3293810	117636.1	358892
**B 7**	this study	53.3	45.7	36	55.28	3290743	91409.5	276479
**B 70**	this study	77.9	72.8	29	54.34	3292819	113545.5	358992
**B 71**	this study	354.9	289.3	26	54.95	3289207	126508	299153
**B 75**	this study	100.6	94.1	32	54.17	3294230	102944.7	276408
**B 76**	this study	80.9	75.7	30	54.38	3294122	109804.1	293096
**B 77**	this study	88.9	83.4	28	54.39	3292795	117599.8	248448
**B 78**	this study	108	101.5	30	53.92	3294011	109800.4	276615
**B 79**	this study	95.6	89.2	28	54.13	3294831	117672.5	221689
**B 8**	this study	165.1	135.7	35	55.07	3290919	94026.3	193054
**B 80**	this study	90	83.9	31	54.76	3294084	106260.8	221721
**B 81**	this study	88.2	82.5	28	54.37	3294086	117645.9	358944
**B 82**	this study	106.8	99.8	29	54.26	3293328	113563	338430
**B 85**	this study	306.6	247.8	34	54.89	3288657	96725.2	293170
**B 9**	this study	59	59	61	55.58	3294589	54009.7	276453
**ERZ4251120**	2	197.57	71.8	39	54.04	3294689	84479.2	293202
**ERZ4251121**	2	240.93	91.55	37	54.61	3294681	89045.4	250966
**ERZ4251122**	2	249.05	90.14	40	54.74	3294654	82366.4	249474
**ERZ4251123**	2	240.4	89.31	40	54.78	3294729	82368.2	249490
**ERZ4251124**	2	37.83	28.97	54	56.10	3294509	61009.4	105611
**ERZ4251125**	2	363.81	99.96	40	55.04	3294398	82360.0	189528
**ERZ4251126**	2	453.66	99.98	39	55.03	3294399	84471.8	276973
**ERZ4251127**	2	279.25	94.9	48	55.31	3295411	68654.4	190031
**ERZ4251128**	2	218.28	79.54	72	49.56	3469854	48192.4	249442
**ERZ4251129**	2	250.6	91.02	40	54.37	3295098	82377.5	195382
**ERZ4251130**	2	143.73	51.5	41	55.14	3294927	80364.1	249474
**ERZ4251131**	2	173.78	66.09	39	54.64	3295374	84496.8	293271

**ERZ4251132**	2	307.61	100	36	54.57	3294405	91511.3	276638
**ERZ4251133**	2	89.82	67.21	50	54.56	3300840	66016.8	222112
**ERZ4251134**	2	225.2	87.01	31	54.69	3294225	106265.3	252965
**ERZ4251135**	2	155.36	54.99	38	54.59	3289092	86555.1	249439
**ERZ4251136**	2	145.52	55.46	37	54.94	3294338	89036.2	250942
**ERZ4251137**	2	95.32	70.93	65	54.49	3303614	50824.8	249573
**ERZ4251138**	2	152.34	59.58	42	54.86	3294684	78444.9	221686
**ERZ4251139**	2	75.23	55.05	47	55.28	3299249	70196.8	249534
**ERZ4251140**	2	44.83	33.84	58	55.10	3300058	56897.6	195613
**ERZ4251141**	2	275.41	81.79	46	55.58	3295108	71632.8	159983
**ERZ4251142**	2	111.34	41.72	38	54.58	3294584	86699.6	276973
**ERZ4251143**	2	144.26	100	30	54.98	3289007	109633.6	221604
**ERZ4251144**	2	94.67	68.44	30	55.20	3289106	109636.9	293197
**ERZ4251145**	2	135.27	50.02	44	54.48	3290117	74775.4	215396
**ERZ4251146**	2	227.24	85.53	37	54.96	3294582	89042.8	293202
**ERZ4251147**	2	78.74	56.46	32	55.12	3288845	102776.4	250945
**ERZ4251148**	2	71.17	54.28	31	55.20	3288654	106085.6	221632
**ERZ4251149**	2	208.75	99.96	34	55.50	3288912	96732.7	189512
**ERZ4251150**	2	99.96	78.5	26	55.12	3289158	126506.1	250975
**ERZ4251151**	2	62.07	50.72	29	55.41	3288526	113397.4	189512
**ERZ4251152**	2	187	66.37	44	55.41	3295393	74895.3	221662
**ERZ4251153**	2	33.41	26.12	39	55.37	3289280	84340.5	250975
**ERZ4251154**	2	42.92	32.19	37	55.72	3288421	88876.2	186499
**ERZ4251155**	2	168.04	57.37	39	55.23	3294182	84466.2	249442
**ERZ4251156**	2	149.34	52.12	42	55.13	3295521	78464.8	249434
**ERZ4251157**	2	55.59	31.95	37	55.17	3288372	88874.9	178965
**ERZ4251158**	2	51.87	34.5	34	55.36	3287913	96703.3	178970
**ERZ4251159**	2	45.5	35.06	34	55.43	3288434	96718.6	189512
**ERZ4251160**	2	48.95	37.91	34	55.28	3288755	96728.1	221673
**ERZ4251161**	2	205.02	99.97	33	55.61	3289414	99679.2	221689
**ERZ4251162**	2	20.46	16.74	40	55.32	3289155	82228.9	276509
**ERZ4251163**	2	77.81	51.3	36	55.19	3288839	91356.6	189512

**ERZ4251164**	2	77.49	60.09	35	55.30	3289215	93977.6	189512
**ERZ4251165**	2	96.9	70.16	40	55.69	3289067	82226.7	189512
**ERZ4251166**	2	203.33	63.38	53	54.94	3296508	62198.3	195417
**ERZ4251167**	2	191.95	50.22	54	55.84	3296671	61049.5	127420
**ERZ4251168**	2	303.7	75.87	43	55.46	3294959	76627.0	195466
**ERZ4251169**	2	222.94	68.4	42	55.56	3295671	78468.4	249474
**ERZ4251170**	2	311.26	92.26	49	55.72	3295527	67255.7	138227
**ERZ4251171**	2	296.29	91.33	43	55.48	3295112	76630.5	189528
**ERZ4251172**	2	380.66	100	45	55.41	3295734	73238.5	193063
**ERZ4251173**	2	182.07	54.54	54	55.85	3295446	61026.8	138227
**ERZ4251174**	2	240.38	70.97	47	55.60	3295968	70127.0	159983
**ERZ4251175**	2	210.31	61.92	50	55.85	3295857	65917.1	138227
**ERZ4251176**	2	243.39	70.63	48	55.26	3295690	68660.2	195434
**ERZ4251177**	2	198.24	60.1	47	55.70	3295026	70106.9	158473
**ERZ4251178**	2	172.96	52.63	47	55.86	3295695	70121.2	195429
**ERZ4251179**	2	324.95	100.15	35	55.59	3289274	93979.3	250969
**ERZ4251180**	2	277.26	100.05	34	55.05	3289065	96737.2	293202
**ERZ4251181**	2	334.1	100.2	35	55.36	3289088	93973.9	249434
**ERZ4251182**	2	372.6	100.2	89	53.57	3313041	37225.2	296694
**ERZ4251183**	2	309.47	100.12	33	55.37	3289038	99667.8	293199
**ERZ4251184**	2	140.58	50.06	45	55.12	3295147	73225.5	195431
**ERZ4251185**	2	76.46	29.45	45	55.51	3294380	73208.4	195437
**ERZ4251186**	2	112.96	39.5	40	55.83	3294002	82350.1	249474
**ERZ4251187**	2	99.58	36.17	43	55.27	3294317	76612.0	160255
**ERZ4251188**	2	101.43	35.32	72	54.32	3300180	45835.8	195454
**ERZ4251189**	2	132.75	47.77	41	55.37	3294085	80343.5	160329
**ERZ4251190**	2	148.84	51.93	43	55.43	3294622	76619.1	163432
**ERZ4251191**	2	112.98	40.63	51	55.70	3294669	64601.4	138227
**ERZ4251192**	2	107.63	39.07	42	55.98	3294153	78432.2	164417
**ERZ4251193**	2	128.42	46.65	42	55.29	3294416	78438.5	250966
**ERZ4251194**	2	123.85	46.36	41	55.24	3294302	80348.8	195445
**ERZ4251195**	2	138.84	50.05	46	55.64	3294754	71625.1	182651

**ERZ4251196**	2	89.92	32.26	48	55.63	3294369	68632.7	173853
**ERZ4251197**	2	99.19	38.9	40	55.25	3294499	82362.5	249432
**ERZ4251198**	2	125.9	91.54	41	55.14	3298719	80456.6	251042
**ERZ4251199**	2	102.43	42.02	41	55.37	3294783	80360.6	195429
**ERZ4251200**	2	145.49	45.49	46	55.59	3294081	71610.5	221676
**ERZ4251201**	2	158.93	58.66	43	55.10	3295048	76629.0	249490
**ERZ4251202**	2	130.6	42.92	46	55.48	3294391	71617.2	174619
**ERZ4251203**	2	242.19	75.97	44	55.57	3294236	74869.0	189528
**ERZ4251204**	2	269.22	93.05	38	55.00	3294501	86697.4	276966
**ERZ4251205**	2	267.6	84.31	36	55.14	3294015	91500.4	250942
**ERZ4251206**	2	161.54	49.86	52	55.66	3294611	63357.9	138227
**ERZ4251207**	2	157.09	44.73	59	55.81	3294927	55846.2	127420
**ERZ4251208**	2	190.23	59.4	43	55.56	3294074	76606.4	174617
**ERZ4251209**	2	207.13	68.33	43	55.30	3294316	76612.0	195452
**ERZ4251210**	2	159.21	45.88	57	55.85	3294511	57798.4	122045
**ERZ4251211**	2	121.05	57.4	36	54.83	3294291	91508.1	293257
**ERZ4251212**	2	115.29	57.25	39	55.02	3294332	84470.1	276495
**SRR11178063**	1	64.32	44.49	60	55.68	3291288	54854.8	155967
**SRR11178065**	1	35.75	24.07	100	55.59	3289408	32894.1	72518
**SRR11178066**	1	50.87	36.02	78	55.29	3289834	42177.4	110978
**SRR11178067**	1	46.03	32.29	76	56.23	3291077	43303.6	90560
**SRR11178068**	1	81.43	55.43	61	55.70	3289874	53932.4	122769
**SRR11178069**	1	65.33	44.39	62	55.96	3291499	53088.7	138308
**SRR11178070**	1	54.31	39.17	66	55.48	3290905	49862.2	136808
**SRR11178071**	1	76.23	49.69	63	55.63	3292363	52259.7	118655
**SRR11178072**	1	48.4	37.47	32	55.22	3288336	102760.5	250945
**SRR11178073**	1	30.36	19.68	89	55.91	3287574	36939.0	67600
**SRR11178074**	1	62.19	42.3	120	55.90	3299864	27498.9	62315
**SRR11178075**	1	119.04	81.15	52	55.83	3290742	63283.5	128823
**SRR11178076**	1	24.18	14.65	136	56.08	3289323	24186.2	42852
**SRR11178077**	1	45.47	30.59	72	55.65	3291798	45719.4	90881
**SRR11178078**	1	32.53	22.32	88	55.66	3290726	37394.6	90858

**SRR11178079**	1	36.67	24.08	102	55.71	3289325	32248.3	65804
**SRR11178080**	1	44.67	28.63	87	55.78	3288671	37800.8	79350
**SRR11178081**	1	58.14	39.7	59	55.73	3291166	55782.5	128863
**SRR11178083**	1	65.42	40.96	53	55.77	3291139	62097.0	136010
**SRR11178084**	1	79.46	54.33	60	55.96	3293229	54887.2	138308
**SRR11178085**	1	89.66	60.33	47	55.37	3291544	70032.9	155716
**SRR11178086**	1	63.06	49.61	34	55.12	3288893	96732.1	182669
**SRR11178087**	1	56.11	36.67	67	55.93	3291104	49121.0	116512
**SRR11178088**	1	61.24	48.07	29	55.03	3288572	113399.0	276473
**SRR11178089**	1	37.51	19.94	107	55.86	3291402	30760.8	61572

References

**1**	Rabinowitz, Peter, et al. “Whole Genome Sequence Analysis of Brucella Melitensis Phylogeny and Virulence Factors.” Microbiology Research, vol. 12, no. 3, Aug. 2021, pp. 698–710. DOI.org (Crossref), https://doi.org/10.3390/microbiolres12030050.
**2**	Zilberman, Bar, et al. “Genomic Epidemiology of Clinical Brucella Melitensis Isolates from Southern Israel.” Microorganisms, vol. 10, no. 2, Jan. 2022, p. 238. DOI.org (Crossref), https://doi.org/10.3390/microorganisms10020238.

Table S3. *In silico* sequence typing results for 230 Israeli *B. melitensis* isolates.

				MLST								
ID	Reference	scheme	ST	allelic profile								
**B 10**	this study	brucella	8	gap(3)	aroA(2)	glk(3)	dnaK(2)	gyrB(1)	trpE(5)	cobQ(3)	int_hyp(2)	omp25(8)
**B 103**	this study	brucella	8	gap(3)	aroA(2)	glk(3)	dnaK(2)	gyrB(1)	trpE(5)	cobQ(3)	int_hyp(2)	omp25(8)
**B 104**	this study	brucella	8	gap(3)	aroA(2)	glk(3)	dnaK(2)	gyrB(1)	trpE(5)	cobQ(3)	int_hyp(2)	omp25(8)
**B 106**	this study	brucella	8	gap(3)	aroA(2)	glk(3)	dnaK(2)	gyrB(1)	trpE(5)	cobQ(3)	int_hyp(2)	omp25(8)
**B 107**	this study	brucella	8	gap(3)	aroA(2)	glk(3)	dnaK(2)	gyrB(1)	trpE(5)	cobQ(3)	int_hyp(2)	omp25(8)
**B 108**	this study	brucella	8	gap(3)	aroA(2)	glk(3)	dnaK(2)	gyrB(1)	trpE(5)	cobQ(3)	int_hyp(2)	omp25(8)
**B 109**	this study	brucella	8	gap(3)	aroA(2)	glk(3)	dnaK(2)	gyrB(1)	trpE(5)	cobQ(3)	int_hyp(2)	omp25(8)
**B 11**	this study	brucella	8	gap(3)	aroA(2)	glk(3)	dnaK(2)	gyrB(1)	trpE(5)	cobQ(3)	int_hyp(2)	omp25(8)
**B 110**	this study	brucella	8	gap(3)	aroA(2)	glk(3)	dnaK(2)	gyrB(1)	trpE(5)	cobQ(3)	int_hyp(2)	omp25(8)
**B 111**	this study	brucella	8	gap(3)	aroA(2)	glk(3)	dnaK(2)	gyrB(1)	trpE(5)	cobQ(3)	int_hyp(2)	omp25(8)
**B 112**	this study	brucella	8	gap(3)	aroA(2)	glk(3)	dnaK(2)	gyrB(1)	trpE(5)	cobQ(3)	int_hyp(2)	omp25(8)
**B 113**	this study	brucella	8	gap(3)	aroA(2)	glk(3)	dnaK(2)	gyrB(1)	trpE(5)	cobQ(3)	int_hyp(2)	omp25(8)
**B 114**	this study	brucella	8	gap(3)	aroA(2)	glk(3)	dnaK(2)	gyrB(1)	trpE(5)	cobQ(3)	int_hyp(2)	omp25(8)
**B 115**	this study	brucella	8	gap(3)	aroA(2)	glk(3)	dnaK(2)	gyrB(1)	trpE(5)	cobQ(3)	int_hyp(2)	omp25(8)
**B 116**	this study	brucella	8	gap(3)	aroA(2)	glk(3)	dnaK(2)	gyrB(1)	trpE(5)	cobQ(3)	int_hyp(2)	omp25(8)
**B 117**	this study	brucella	8	gap(3)	aroA(2)	glk(3)	dnaK(2)	gyrB(1)	trpE(5)	cobQ(3)	int_hyp(2)	omp25(8)
**B 118**	this study	brucella	-	gap(3)	aroA(2)	glk(3)	dnaK(2)	gyrB(27?)	trpE(5)	cobQ(3)	int_hyp(2)	omp25(8)
**B 119**	this study	brucella	8	gap(3)	aroA(2)	glk(3)	dnaK(2)	gyrB(1)	trpE(5)	cobQ(3)	int_hyp(2)	omp25(8)
**B 12**	this study	brucella	8	gap(3)	aroA(2)	glk(3)	dnaK(2)	gyrB(1)	trpE(5)	cobQ(3)	int_hyp(2)	omp25(8)
**B 120**	this study	brucella	8	gap(3)	aroA(2)	glk(3)	dnaK(2)	gyrB(1)	trpE(5)	cobQ(3)	int_hyp(2)	omp25(8)
**B 121**	this study	brucella	8	gap(3)	aroA(2)	glk(3)	dnaK(2)	gyrB(1)	trpE(5)	cobQ(3)	int_hyp(2)	omp25(8)
**B 122**	this study	brucella	8	gap(3)	aroA(2)	glk(3)	dnaK(2)	gyrB(1)	trpE(5)	cobQ(3)	int_hyp(2)	omp25(8)
**B 123**	this study	brucella	8	gap(3)	aroA(2)	glk(3)	dnaK(2)	gyrB(1)	trpE(5)	cobQ(3)	int_hyp(2)	omp25(8)
**B 124**	this study	brucella	-	gap(3)	aroA(2)	glk(3)	dnaK(2)	gyrB(1)	trpE(5)	cobQ(9?)	int_hyp(2)	omp25(8)
**B 125**	this study	brucella	8	gap(3)	aroA(2)	glk(3)	dnaK(2)	gyrB(1)	trpE(5)	cobQ(3)	int_hyp(2)	omp25(8)
**B 126**	this study	brucella	8	gap(3)	aroA(2)	glk(3)	dnaK(2)	gyrB(1)	trpE(5)	cobQ(3)	int_hyp(2)	omp25(8)
**B 127**	this study	brucella	8	gap(3)	aroA(2)	glk(3)	dnaK(2)	gyrB(1)	trpE(5)	cobQ(3)	int_hyp(2)	omp25(8)
**B 128**	this study	brucella	8	gap(3)	aroA(2)	glk(3)	dnaK(2)	gyrB(1)	trpE(5)	cobQ(3)	int_hyp(2)	omp25(8)
**B 129**	this study	brucella	8	gap(3)	aroA(2)	glk(3)	dnaK(2)	gyrB(1)	trpE(5)	cobQ(3)	int_hyp(2)	omp25(8)

**B 13**	this study	brucella	8	gap(3)	aroA(2)	glk(3)	dnaK(2)	gyrB(1)	trpE(5)	cobQ(3)	int_hyp(2)	omp25(8)
**B 130**	this study	brucella	8	gap(3)	aroA(2)	glk(3)	dnaK(2)	gyrB(1)	trpE(5)	cobQ(3)	int_hyp(2)	omp25(8)
**B 131**	this study	brucella	8	gap(3)	aroA(2)	glk(3)	dnaK(2)	gyrB(1)	trpE(5)	cobQ(3)	int_hyp(2)	omp25(8)
**B 132**	this study	brucella	8	gap(3)	aroA(2)	glk(3)	dnaK(2)	gyrB(1)	trpE(5)	cobQ(3)	int_hyp(2)	omp25(8)
**B 133**	this study	brucella	8	gap(3)	aroA(2)	glk(3)	dnaK(2)	gyrB(1)	trpE(5)	cobQ(3)	int_hyp(2)	omp25(8)
**B 134**	this study	brucella	8	gap(3)	aroA(2)	glk(3)	dnaK(2)	gyrB(1)	trpE(5)	cobQ(3)	int_hyp(2)	omp25(8)
**B 135**	this study	brucella	8	gap(3)	aroA(2)	glk(3)	dnaK(2)	gyrB(1)	trpE(5)	cobQ(3)	int_hyp(2)	omp25(8)
**B 137**	this study	brucella	8	gap(3)	aroA(2)	glk(3)	dnaK(2)	gyrB(1)	trpE(5)	cobQ(3)	int_hyp(2)	omp25(8)
**B 138**	this study	brucella	8	gap(3)	aroA(2)	glk(3)	dnaK(2)	gyrB(1)	trpE(5)	cobQ(3)	int_hyp(2)	omp25(8)
**B 139**	this study	brucella	8	gap(3)	aroA(2)	glk(3)	dnaK(2)	gyrB(1)	trpE(5)	cobQ(3)	int_hyp(2)	omp25(8)
**B 14**	this study	brucella	8	gap(3)	aroA(2)	glk(3)	dnaK(2)	gyrB(1)	trpE(5)	cobQ(3)	int_hyp(2)	omp25(8)
**B 140**	this study	brucella	8	gap(3)	aroA(2)	glk(3)	dnaK(2)	gyrB(1)	trpE(5)	cobQ(3)	int_hyp(2)	omp25(8)
**B 141**	this study	brucella	8	gap(3)	aroA(2)	glk(3)	dnaK(2)	gyrB(1)	trpE(5)	cobQ(3)	int_hyp(2)	omp25(8)
**B 142**	this study	brucella	8	gap(3)	aroA(2)	glk(3)	dnaK(2)	gyrB(1)	trpE(5)	cobQ(3)	int_hyp(2)	omp25(8)
**B 143**	this study	brucella	8	gap(3)	aroA(2)	glk(3)	dnaK(2)	gyrB(1)	trpE(5)	cobQ(3)	int_hyp(2)	omp25(8)
**B 144**	this study	brucella	8	gap(3)	aroA(2)	glk(3)	dnaK(2)	gyrB(1)	trpE(5)	cobQ(3)	int_hyp(2)	omp25(8)
**B 145**	this study	brucella	8	gap(3)	aroA(2)	glk(3)	dnaK(2)	gyrB(1)	trpE(5)	cobQ(3)	int_hyp(2)	omp25(8)
**B 146**	this study	brucella	8	gap(3)	aroA(2)	glk(3)	dnaK(2)	gyrB(1)	trpE(5)	cobQ(3)	int_hyp(2)	omp25(8)
**B 148**	this study	brucella	-	gap(12?)	aroA(2)	glk(3)	dnaK(2)	gyrB(1)	trpE(5)	cobQ(3)	int_hyp(2)	omp25(8)
**B 149**	this study	brucella	8	gap(3)	aroA(2)	glk(3)	dnaK(2)	gyrB(1)	trpE(5)	cobQ(3)	int_hyp(2)	omp25(8)
**B 15**	this study	brucella	8	gap(3)	aroA(2)	glk(3)	dnaK(2)	gyrB(1)	trpE(5)	cobQ(3)	int_hyp(2)	omp25(8)
**B 150**	this study	brucella	8	gap(3)	aroA(2)	glk(3)	dnaK(2)	gyrB(1)	trpE(5)	cobQ(3)	int_hyp(2)	omp25(8)
**B 151**	this study	brucella	8	gap(3)	aroA(2)	glk(3)	dnaK(2)	gyrB(1)	trpE(5)	cobQ(3)	int_hyp(2)	omp25(8)
**B 152**	this study	brucella	8	gap(3)	aroA(2)	glk(3)	dnaK(2)	gyrB(1)	trpE(5)	cobQ(3)	int_hyp(2)	omp25(8)
**B 153**	this study	brucella	-	gap(3?)	aroA(16?)	glk(3)	dnaK(2)	gyrB(1)	trpE(5)	cobQ(27?)	int_hyp(2)	omp25(8)
**B 154**	this study	brucella	8	gap(3)	aroA(2)	glk(3)	dnaK(2)	gyrB(1)	trpE(5)	cobQ(3)	int_hyp(2)	omp25(8)
**B 155**	this study	brucella	8	gap(3)	aroA(2)	glk(3)	dnaK(2)	gyrB(1)	trpE(5)	cobQ(3)	int_hyp(2)	omp25(8)
**B 156**	this study	brucella	8	gap(3)	aroA(2)	glk(3)	dnaK(2)	gyrB(1)	trpE(5)	cobQ(3)	int_hyp(2)	omp25(8)
**B 157**	this study	brucella	8	gap(3)	aroA(2)	glk(3)	dnaK(2)	gyrB(1)	trpE(5)	cobQ(3)	int_hyp(2)	omp25(8)
**B 158**	this study	brucella	8	gap(3)	aroA(2)	glk(3)	dnaK(2)	gyrB(1)	trpE(5)	cobQ(3)	int_hyp(2)	omp25(8)
**B 159**	this study	brucella	8	gap(3)	aroA(2)	glk(3)	dnaK(2)	gyrB(1)	trpE(5)	cobQ(3)	int_hyp(2)	omp25(8)

**B 16**	this study	brucella	8	gap(3)	aroA(2)	glk(3)	dnaK(2)	gyrB(1)	trpE(5)	cobQ(3)	int_hyp(2)	omp25(8)
**B 160**	this study	brucella	8	gap(3)	aroA(2)	glk(3)	dnaK(2)	gyrB(1)	trpE(5)	cobQ(3)	int_hyp(2)	omp25(8)
**B 161**	this study	brucella	8	gap(3)	aroA(2)	glk(3)	dnaK(2)	gyrB(1)	trpE(5)	cobQ(3)	int_hyp(2)	omp25(8)
**B 162**	this study	brucella	8	gap(3)	aroA(2)	glk(3)	dnaK(2)	gyrB(1)	trpE(5)	cobQ(3)	int_hyp(2)	omp25(8)
**B 17**	this study	brucella	8	gap(3)	aroA(2)	glk(3)	dnaK(2)	gyrB(1)	trpE(5)	cobQ(3)	int_hyp(2)	omp25(8)
**B 18**	this study	brucella	8	gap(3)	aroA(2)	glk(3)	dnaK(2)	gyrB(1)	trpE(5)	cobQ(3)	int_hyp(2)	omp25(8)
**B 19**	this study	brucella	8	gap(3)	aroA(2)	glk(3)	dnaK(2)	gyrB(1)	trpE(5)	cobQ(3)	int_hyp(2)	omp25(8)
**B 2**	this study	brucella	8	gap(3)	aroA(2)	glk(3)	dnaK(2)	gyrB(1)	trpE(5)	cobQ(3)	int_hyp(2)	omp25(8)
**B 20**	this study	brucella	8	gap(3)	aroA(2)	glk(3)	dnaK(2)	gyrB(1)	trpE(5)	cobQ(3)	int_hyp(2)	omp25(8)
**B 21**	this study	brucella	8	gap(3)	aroA(2)	glk(3)	dnaK(2)	gyrB(1)	trpE(5)	cobQ(3)	int_hyp(2)	omp25(8)
**B 22**	this study	brucella	8	gap(3)	aroA(2)	glk(3)	dnaK(2)	gyrB(1)	trpE(5)	cobQ(3)	int_hyp(2)	omp25(8)
**B 23**	this study	brucella	8	gap(3)	aroA(2)	glk(3)	dnaK(2)	gyrB(1)	trpE(5)	cobQ(3)	int_hyp(2)	omp25(8)
**B 24**	this study	brucella	8	gap(3)	aroA(2)	glk(3)	dnaK(2)	gyrB(1)	trpE(5)	cobQ(3)	int_hyp(2)	omp25(8)
**B 25**	this study	brucella	8	gap(3)	aroA(2)	glk(3)	dnaK(2)	gyrB(1)	trpE(5)	cobQ(3)	int_hyp(2)	omp25(8)
**B 32**	this study	brucella	8	gap(3)	aroA(2)	glk(3)	dnaK(2)	gyrB(1)	trpE(5)	cobQ(3)	int_hyp(2)	omp25(8)
**B 35**	this study	brucella	8	gap(3)	aroA(2)	glk(3)	dnaK(2)	gyrB(1)	trpE(5)	cobQ(3)	int_hyp(2)	omp25(8)
**B 36**	this study	brucella	8	gap(3)	aroA(2)	glk(3)	dnaK(2)	gyrB(1)	trpE(5)	cobQ(3)	int_hyp(2)	omp25(8)
**B 37**	this study	brucella	8	gap(3)	aroA(2)	glk(3)	dnaK(2)	gyrB(1)	trpE(5)	cobQ(3)	int_hyp(2)	omp25(8)
**B 38**	this study	brucella	8	gap(3)	aroA(2)	glk(3)	dnaK(2)	gyrB(1)	trpE(5)	cobQ(3)	int_hyp(2)	omp25(8)
**B 39**	this study	brucella	8	gap(3)	aroA(2)	glk(3)	dnaK(2)	gyrB(1)	trpE(5)	cobQ(3)	int_hyp(2)	omp25(8)
**B 40**	this study	brucella	8	gap(3)	aroA(2)	glk(3)	dnaK(2)	gyrB(1)	trpE(5)	cobQ(3)	int_hyp(2)	omp25(8)
**B 47**	this study	brucella	8	gap(3)	aroA(2)	glk(3)	dnaK(2)	gyrB(1)	trpE(5)	cobQ(3)	int_hyp(2)	omp25(8)
**B 48**	this study	brucella	8	gap(3)	aroA(2)	glk(3)	dnaK(2)	gyrB(1)	trpE(5)	cobQ(3)	int_hyp(2)	omp25(8)
**B 5**	this study	brucella	8	gap(3)	aroA(2)	glk(3)	dnaK(2)	gyrB(1)	trpE(5)	cobQ(3)	int_hyp(2)	omp25(8)
**B 50**	this study	brucella	8	gap(3)	aroA(2)	glk(3)	dnaK(2)	gyrB(1)	trpE(5)	cobQ(3)	int_hyp(2)	omp25(8)
**B 51**	this study	brucella	8	gap(3)	aroA(2)	glk(3)	dnaK(2)	gyrB(1)	trpE(5)	cobQ(3)	int_hyp(2)	omp25(8)
**B 52**	this study	brucella	8	gap(3)	aroA(2)	glk(3)	dnaK(2)	gyrB(1)	trpE(5)	cobQ(3)	int_hyp(2)	omp25(8)
**B 53**	this study	brucella	8	gap(3)	aroA(2)	glk(3)	dnaK(2)	gyrB(1)	trpE(5)	cobQ(3)	int_hyp(2)	omp25(8)
**B 54**	this study	brucella	8	gap(3)	aroA(2)	glk(3)	dnaK(2)	gyrB(1)	trpE(5)	cobQ(3)	int_hyp(2)	omp25(8)
**B 55**	this study	brucella	8	gap(3)	aroA(2)	glk(3)	dnaK(2)	gyrB(1)	trpE(5)	cobQ(3)	int_hyp(2)	omp25(8)
**B 56**	this study	brucella	8	gap(3)	aroA(2)	glk(3)	dnaK(2)	gyrB(1)	trpE(5)	cobQ(3)	int_hyp(2)	omp25(8)
**B 57**	this study	brucella	8	gap(3)	aroA(2)	glk(3)	dnaK(2)	gyrB(1)	trpE(5)	cobQ(3)	int_hyp(2)	omp25(8)

**B 6**	this study	brucella	8	gap(3)	aroA(2)	glk(3)	dnaK(2)	gyrB(1)	trpE(5)	cobQ(3)	int_hyp(2)	omp25(8)
**B 65**	this study	brucella	8	gap(3)	aroA(2)	glk(3)	dnaK(2)	gyrB(1)	trpE(5)	cobQ(3)	int_hyp(2)	omp25(8)
**B 66**	this study	brucella	8	gap(3)	aroA(2)	glk(3)	dnaK(2)	gyrB(1)	trpE(5)	cobQ(3)	int_hyp(2)	omp25(8)
**B 67**	this study	brucella	8	gap(3)	aroA(2)	glk(3)	dnaK(2)	gyrB(1)	trpE(5)	cobQ(3)	int_hyp(2)	omp25(8)
**B 68**	this study	brucella	-	gap(3)	aroA(2)	glk(35)	dnaK(2)	gyrB(1)	trpE(5)	cobQ(3)	int_hyp(2)	omp25(8)
**B 69**	this study	brucella	8	gap(3)	aroA(2)	glk(3)	dnaK(2)	gyrB(1)	trpE(5)	cobQ(3)	int_hyp(2)	omp25(8)
**B 7**	this study	brucella	8	gap(3)	aroA(2)	glk(3)	dnaK(2)	gyrB(1)	trpE(5)	cobQ(3)	int_hyp(2)	omp25(8)
**B 70**	this study	brucella	8	gap(3)	aroA(2)	glk(3)	dnaK(2)	gyrB(1)	trpE(5)	cobQ(3)	int_hyp(2)	omp25(8)
**B 71**	this study	brucella	8	gap(3)	aroA(2)	glk(3)	dnaK(2)	gyrB(1)	trpE(5)	cobQ(3)	int_hyp(2)	omp25(8)
**B 75**	this study	brucella	-	gap(3)	aroA(2)	glk(35)	dnaK(2)	gyrB(1)	trpE(5)	cobQ(3)	int_hyp(2)	omp25(8)
**B 76**	this study	brucella	8	gap(3)	aroA(2)	glk(3)	dnaK(2)	gyrB(1)	trpE(5)	cobQ(3)	int_hyp(2)	omp25(8)
**B 77**	this study	brucella	8	gap(3)	aroA(2)	glk(3)	dnaK(2)	gyrB(1)	trpE(5)	cobQ(3)	int_hyp(2)	omp25(8)
**B 78**	this study	brucella	-	gap(3)	aroA(2)	glk(35)	dnaK(2)	gyrB(1)	trpE(5)	cobQ(3)	int_hyp(2)	omp25(8)
**B 79**	this study	brucella	8	gap(3)	aroA(2)	glk(3)	dnaK(2)	gyrB(1)	trpE(5)	cobQ(3)	int_hyp(2)	omp25(8)
**B 8**	this study	brucella	8	gap(3)	aroA(2)	glk(3)	dnaK(2)	gyrB(1)	trpE(5)	cobQ(3)	int_hyp(2)	omp25(8)
**B 80**	this study	brucella	8	gap(3)	aroA(2)	glk(3)	dnaK(2)	gyrB(1)	trpE(5)	cobQ(3)	int_hyp(2)	omp25(8)
**B 81**	this study	brucella	8	gap(3)	aroA(2)	glk(3)	dnaK(2)	gyrB(1)	trpE(5)	cobQ(3)	int_hyp(2)	omp25(8)
**B 82**	this study	brucella	8	gap(3)	aroA(2)	glk(3)	dnaK(2)	gyrB(1)	trpE(5)	cobQ(3)	int_hyp(2)	omp25(8)
**B 85**	this study	brucella	8	gap(3)	aroA(2)	glk(3)	dnaK(2)	gyrB(1)	trpE(5)	cobQ(3)	int_hyp(2)	omp25(8)
**B 9**	this study	brucella	8	gap(3)	aroA(2)	glk(3)	dnaK(2)	gyrB(1)	trpE(5)	cobQ(3)	int_hyp(2)	omp25(8)
**ERZ4251120**	2	brucella	8	gap(3)	aroA(2)	glk(3)	dnaK(2)	gyrB(1)	trpE(5)	cobQ(3)	int_hyp(2)	omp25(8)
**ERZ4251121**	2	brucella	8	gap(3)	aroA(2)	glk(3)	dnaK(2)	gyrB(1)	trpE(5)	cobQ(3)	int_hyp(2)	omp25(8)
**ERZ4251122**	2	brucella	8	gap(3)	aroA(2)	glk(3)	dnaK(2)	gyrB(1)	trpE(5)	cobQ(3)	int_hyp(2)	omp25(8)
**ERZ4251123**	2	brucella	8	gap(3)	aroA(2)	glk(3)	dnaK(2)	gyrB(1)	trpE(5)	cobQ(3)	int_hyp(2)	omp25(8)
**ERZ4251124**	2	brucella	8	gap(3)	aroA(2)	glk(3)	dnaK(2)	gyrB(1)	trpE(5)	cobQ(3)	int_hyp(2)	omp25(8)
**ERZ4251125**	2	brucella	8	gap(3)	aroA(2)	glk(3)	dnaK(2)	gyrB(1)	trpE(5)	cobQ(3)	int_hyp(2)	omp25(8)
**ERZ4251126**	2	brucella	8	gap(3)	aroA(2)	glk(3)	dnaK(2)	gyrB(1)	trpE(5)	cobQ(3)	int_hyp(2)	omp25(8)
**ERZ4251127**	2	brucella	8	gap(3)	aroA(2)	glk(3)	dnaK(2)	gyrB(1)	trpE(5)	cobQ(3)	int_hyp(2)	omp25(8)
**ERZ4251128**	2	brucella	8	gap(3)	aroA(2)	glk(3)	dnaK(2)	gyrB(1)	trpE(5)	cobQ(3)	int_hyp(2)	omp25(8)
**ERZ4251129**	2	brucella	8	gap(3)	aroA(2)	glk(3)	dnaK(2)	gyrB(1)	trpE(5)	cobQ(3)	int_hyp(2)	omp25(8)
**ERZ4251130**	2	brucella	8	gap(3)	aroA(2)	glk(3)	dnaK(2)	gyrB(1)	trpE(5)	cobQ(3)	int_hyp(2)	omp25(8)
**ERZ4251131**	2	brucella	8	gap(3)	aroA(2)	glk(3)	dnaK(2)	gyrB(1)	trpE(5)	cobQ(3)	int_hyp(2)	omp25(8)

**ERZ4251132**	2	brucella	8	gap(3)	aroA(2)	glk(3)	dnaK(2)	gyrB(1)	trpE(5)	cobQ(3)	int_hyp(2)	omp25(8)
**ERZ4251133**	2	brucella	8	gap(3)	aroA(2)	glk(3)	dnaK(2)	gyrB(1)	trpE(5)	cobQ(3)	int_hyp(2)	omp25(8)
**ERZ4251134**	2	brucella	8	gap(3)	aroA(2)	glk(3)	dnaK(2)	gyrB(1)	trpE(5)	cobQ(3)	int_hyp(2)	omp25(8)
**ERZ4251135**	2	brucella	8	gap(3)	aroA(2)	glk(3)	dnaK(2)	gyrB(1)	trpE(5)	cobQ(3)	int_hyp(2)	omp25(8)
**ERZ4251136**	2	brucella	8	gap(3)	aroA(2)	glk(3)	dnaK(2)	gyrB(1)	trpE(5)	cobQ(3)	int_hyp(2)	omp25(8)
**ERZ4251137**	2	brucella	8	gap(3)	aroA(2)	glk(3)	dnaK(2)	gyrB(1)	trpE(5)	cobQ(3)	int_hyp(2)	omp25(8)
**ERZ4251138**	2	brucella	8	gap(3)	aroA(2)	glk(3)	dnaK(2)	gyrB(1)	trpE(5)	cobQ(3)	int_hyp(2)	omp25(8)
**ERZ4251139**	2	brucella	8	gap(3)	aroA(2)	glk(3)	dnaK(2)	gyrB(1)	trpE(5)	cobQ(3)	int_hyp(2)	omp25(8)
**ERZ4251140**	2	brucella	8	gap(3)	aroA(2)	glk(3)	dnaK(2)	gyrB(1)	trpE(5)	cobQ(3)	int_hyp(2)	omp25(8)
**ERZ4251141**	2	brucella	8	gap(3)	aroA(2)	glk(3)	dnaK(2)	gyrB(1)	trpE(5)	cobQ(3)	int_hyp(2)	omp25(8)
**ERZ4251142**	2	brucella	8	gap(3)	aroA(2)	glk(3)	dnaK(2)	gyrB(1)	trpE(5)	cobQ(3)	int_hyp(2)	omp25(8)
**ERZ4251143**	2	brucella	8	gap(3)	aroA(2)	glk(3)	dnaK(2)	gyrB(1)	trpE(5)	cobQ(3)	int_hyp(2)	omp25(8)
**ERZ4251144**	2	brucella	8	gap(3)	aroA(2)	glk(3)	dnaK(2)	gyrB(1)	trpE(5)	cobQ(3)	int_hyp(2)	omp25(8)
**ERZ4251145**	2	brucella	8	gap(3)	aroA(2)	glk(3)	dnaK(2)	gyrB(1)	trpE(5)	cobQ(3)	int_hyp(2)	omp25(8)
**ERZ4251146**	2	brucella	8	gap(3)	aroA(2)	glk(3)	dnaK(2)	gyrB(1)	trpE(5)	cobQ(3)	int_hyp(2)	omp25(8)
**ERZ4251147**	2	brucella	8	gap(3)	aroA(2)	glk(3)	dnaK(2)	gyrB(1)	trpE(5)	cobQ(3)	int_hyp(2)	omp25(8)
**ERZ4251148**	2	brucella	8	gap(3)	aroA(2)	glk(3)	dnaK(2)	gyrB(1)	trpE(5)	cobQ(3)	int_hyp(2)	omp25(8)
**ERZ4251149**	2	brucella	8	gap(3)	aroA(2)	glk(3)	dnaK(2)	gyrB(1)	trpE(5)	cobQ(3)	int_hyp(2)	omp25(8)
**ERZ4251150**	2	brucella	8	gap(3)	aroA(2)	glk(3)	dnaK(2)	gyrB(1)	trpE(5)	cobQ(3)	int_hyp(2)	omp25(8)
**ERZ4251151**	2	brucella	8	gap(3)	aroA(2)	glk(3)	dnaK(2)	gyrB(1)	trpE(5)	cobQ(3)	int_hyp(2)	omp25(8)
**ERZ4251152**	2	brucella	8	gap(3)	aroA(2)	glk(3)	dnaK(2)	gyrB(1)	trpE(5)	cobQ(3)	int_hyp(2)	omp25(8)
**ERZ4251153**	2	brucella	8	gap(3)	aroA(2)	glk(3)	dnaK(2)	gyrB(1)	trpE(5)	cobQ(3)	int_hyp(2)	omp25(8)
**ERZ4251154**	2	brucella	8	gap(3)	aroA(2)	glk(3)	dnaK(2)	gyrB(1)	trpE(5)	cobQ(3)	int_hyp(2)	omp25(8)
**ERZ4251155**	2	brucella	8	gap(3)	aroA(2)	glk(3)	dnaK(2)	gyrB(1)	trpE(5)	cobQ(3)	int_hyp(2)	omp25(8)
**ERZ4251156**	2	brucella	8	gap(3)	aroA(2)	glk(3)	dnaK(2)	gyrB(1)	trpE(5)	cobQ(3)	int_hyp(2)	omp25(8)
**ERZ4251157**	2	brucella	8	gap(3)	aroA(2)	glk(3)	dnaK(2)	gyrB(1)	trpE(5)	cobQ(3)	int_hyp(2)	omp25(8)
**ERZ4251158**	2	brucella	8	gap(3)	aroA(2)	glk(3)	dnaK(2)	gyrB(1)	trpE(5)	cobQ(3)	int_hyp(2)	omp25(8)
**ERZ4251159**	2	brucella	8	gap(3)	aroA(2)	glk(3)	dnaK(2)	gyrB(1)	trpE(5)	cobQ(3)	int_hyp(2)	omp25(8)
**ERZ4251160**	2	brucella	8	gap(3)	aroA(2)	glk(3)	dnaK(2)	gyrB(1)	trpE(5)	cobQ(3)	int_hyp(2)	omp25(8)
**ERZ4251161**	2	brucella	8	gap(3)	aroA(2)	glk(3)	dnaK(2)	gyrB(1)	trpE(5)	cobQ(3)	int_hyp(2)	omp25(8)
**ERZ4251162**	2	brucella	8	gap(3)	aroA(2)	glk(3)	dnaK(2)	gyrB(1)	trpE(5)	cobQ(3)	int_hyp(2)	omp25(8)
**ERZ4251163**	2	brucella	8	gap(3)	aroA(2)	glk(3)	dnaK(2)	gyrB(1)	trpE(5)	cobQ(3)	int_hyp(2)	omp25(8)

**ERZ4251164**	2	brucella	8	gap(3)	aroA(2)	glk(3)	dnaK(2)	gyrB(1)	trpE(5)	cobQ(3)	int_hyp(2)	omp25(8)
**ERZ4251165**	2	brucella	8	gap(3)	aroA(2)	glk(3)	dnaK(2)	gyrB(1)	trpE(5)	cobQ(3)	int_hyp(2)	omp25(8)
**ERZ4251166**	2	brucella	8	gap(3)	aroA(2)	glk(3)	dnaK(2)	gyrB(1)	trpE(5)	cobQ(3)	int_hyp(2)	omp25(8)
**ERZ4251167**	2	brucella	8	gap(3)	aroA(2)	glk(3)	dnaK(2)	gyrB(1)	trpE(5)	cobQ(3)	int_hyp(2)	omp25(8)
**ERZ4251168**	2	brucella	8	gap(3)	aroA(2)	glk(3)	dnaK(2)	gyrB(1)	trpE(5)	cobQ(3)	int_hyp(2)	omp25(8)
**ERZ4251169**	2	brucella	8	gap(3)	aroA(2)	glk(3)	dnaK(2)	gyrB(1)	trpE(5)	cobQ(3)	int_hyp(2)	omp25(8)
**ERZ4251170**	2	brucella	8	gap(3)	aroA(2)	glk(3)	dnaK(2)	gyrB(1)	trpE(5)	cobQ(3)	int_hyp(2)	omp25(8)
**ERZ4251171**	2	brucella	8	gap(3)	aroA(2)	glk(3)	dnaK(2)	gyrB(1)	trpE(5)	cobQ(3)	int_hyp(2)	omp25(8)
**ERZ4251172**	2	brucella	8	gap(3)	aroA(2)	glk(3)	dnaK(2)	gyrB(1)	trpE(5)	cobQ(3)	int_hyp(2)	omp25(8)
**ERZ4251173**	2	brucella	8	gap(3)	aroA(2)	glk(3)	dnaK(2)	gyrB(1)	trpE(5)	cobQ(3)	int_hyp(2)	omp25(8)
**ERZ4251174**	2	brucella	8	gap(3)	aroA(2)	glk(3)	dnaK(2)	gyrB(1)	trpE(5)	cobQ(3)	int_hyp(2)	omp25(8)
**ERZ4251175**	2	brucella	8	gap(3)	aroA(2)	glk(3)	dnaK(2)	gyrB(1)	trpE(5)	cobQ(3)	int_hyp(2)	omp25(8)
**ERZ4251176**	2	brucella	8	gap(3)	aroA(2)	glk(3)	dnaK(2)	gyrB(1)	trpE(5)	cobQ(3)	int_hyp(2)	omp25(8)
**ERZ4251177**	2	brucella	8	gap(3)	aroA(2)	glk(3)	dnaK(2)	gyrB(1)	trpE(5)	cobQ(3)	int_hyp(2)	omp25(8)
**ERZ4251178**	2	brucella	8	gap(3)	aroA(2)	glk(3)	dnaK(2)	gyrB(1)	trpE(5)	cobQ(3)	int_hyp(2)	omp25(8)
**ERZ4251179**	2	brucella	8	gap(3)	aroA(2)	glk(3)	dnaK(2)	gyrB(1)	trpE(5)	cobQ(3)	int_hyp(2)	omp25(8)
**ERZ4251180**	2	brucella	8	gap(3)	aroA(2)	glk(3)	dnaK(2)	gyrB(1)	trpE(5)	cobQ(3)	int_hyp(2)	omp25(8)
**ERZ4251181**	2	brucella	8	gap(3)	aroA(2)	glk(3)	dnaK(2)	gyrB(1)	trpE(5)	cobQ(3)	int_hyp(2)	omp25(8)
**ERZ4251182**	2	brucella	8	gap(3)	aroA(2)	glk(3)	dnaK(2)	gyrB(1)	trpE(5)	cobQ(3)	int_hyp(2)	omp25(8)
**ERZ4251183**	2	brucella	8	gap(3)	aroA(2)	glk(3)	dnaK(2)	gyrB(1)	trpE(5)	cobQ(3)	int_hyp(2)	omp25(8)
**ERZ4251184**	2	brucella	8	gap(3)	aroA(2)	glk(3)	dnaK(2)	gyrB(1)	trpE(5)	cobQ(3)	int_hyp(2)	omp25(8)
**ERZ4251185**	2	brucella	8	gap(3)	aroA(2)	glk(3)	dnaK(2)	gyrB(1)	trpE(5)	cobQ(3)	int_hyp(2)	omp25(8)
**ERZ4251186**	2	brucella	8	gap(3)	aroA(2)	glk(3)	dnaK(2)	gyrB(1)	trpE(5)	cobQ(3)	int_hyp(2)	omp25(8)
**ERZ4251187**	2	brucella	8	gap(3)	aroA(2)	glk(3)	dnaK(2)	gyrB(1)	trpE(5)	cobQ(3)	int_hyp(2)	omp25(8)
**ERZ4251188**	2	brucella	8	gap(3)	aroA(2)	glk(3)	dnaK(2)	gyrB(1)	trpE(5)	cobQ(3)	int_hyp(2)	omp25(8)
**ERZ4251189**	2	brucella	8	gap(3)	aroA(2)	glk(3)	dnaK(2)	gyrB(1)	trpE(5)	cobQ(3)	int_hyp(2)	omp25(8)
**ERZ4251190**	2	brucella	8	gap(3)	aroA(2)	glk(3)	dnaK(2)	gyrB(1)	trpE(5)	cobQ(3)	int_hyp(2)	omp25(8)
**ERZ4251191**	2	brucella	8	gap(3)	aroA(2)	glk(3)	dnaK(2)	gyrB(1)	trpE(5)	cobQ(3)	int_hyp(2)	omp25(8)
**ERZ4251192**	2	brucella	8	gap(3)	aroA(2)	glk(3)	dnaK(2)	gyrB(1)	trpE(5)	cobQ(3)	int_hyp(2)	omp25(8)
**ERZ4251193**	2	brucella	8	gap(3)	aroA(2)	glk(3)	dnaK(2)	gyrB(1)	trpE(5)	cobQ(3)	int_hyp(2)	omp25(8)
**ERZ4251194**	2	brucella	8	gap(3)	aroA(2)	glk(3)	dnaK(2)	gyrB(1)	trpE(5)	cobQ(3)	int_hyp(2)	omp25(8)
**ERZ4251195**	2	brucella	8	gap(3)	aroA(2)	glk(3)	dnaK(2)	gyrB(1)	trpE(5)	cobQ(3)	int_hyp(2)	omp25(8)

**ERZ4251196**	2	brucella	8	gap(3)	aroA(2)	glk(3)	dnaK(2)	gyrB(1)	trpE(5)	cobQ(3)	int_hyp(2)	omp25(8)
**ERZ4251197**	2	brucella	8	gap(3)	aroA(2)	glk(3)	dnaK(2)	gyrB(1)	trpE(5)	cobQ(3)	int_hyp(2)	omp25(8)
**ERZ4251198**	2	brucella	8	gap(3)	aroA(2)	glk(3)	dnaK(2)	gyrB(1)	trpE(5)	cobQ(3)	int_hyp(2)	omp25(8)
**ERZ4251199**	2	brucella	8	gap(3)	aroA(2)	glk(3)	dnaK(2)	gyrB(1)	trpE(5)	cobQ(3)	int_hyp(2)	omp25(8)
**ERZ4251200**	2	brucella	8	gap(3)	aroA(2)	glk(3)	dnaK(2)	gyrB(1)	trpE(5)	cobQ(3)	int_hyp(2)	omp25(8)
**ERZ4251201**	2	brucella	8	gap(3)	aroA(2)	glk(3)	dnaK(2)	gyrB(1)	trpE(5)	cobQ(3)	int_hyp(2)	omp25(8)
**ERZ4251202**	2	brucella	8	gap(3)	aroA(2)	glk(3)	dnaK(2)	gyrB(1)	trpE(5)	cobQ(3)	int_hyp(2)	omp25(8)
**ERZ4251203**	2	brucella	8	gap(3)	aroA(2)	glk(3)	dnaK(2)	gyrB(1)	trpE(5)	cobQ(3)	int_hyp(2)	omp25(8)
**ERZ4251204**	2	brucella	8	gap(3)	aroA(2)	glk(3)	dnaK(2)	gyrB(1)	trpE(5)	cobQ(3)	int_hyp(2)	omp25(8)
**ERZ4251205**	2	brucella	8	gap(3)	aroA(2)	glk(3)	dnaK(2)	gyrB(1)	trpE(5)	cobQ(3)	int_hyp(2)	omp25(8)
**ERZ4251206**	2	brucella	8	gap(3)	aroA(2)	glk(3)	dnaK(2)	gyrB(1)	trpE(5)	cobQ(3)	int_hyp(2)	omp25(8)
**ERZ4251207**	2	brucella	8	gap(3)	aroA(2)	glk(3)	dnaK(2)	gyrB(1)	trpE(5)	cobQ(3)	int_hyp(2)	omp25(8)
**ERZ4251208**	2	brucella	8	gap(3)	aroA(2)	glk(3)	dnaK(2)	gyrB(1)	trpE(5)	cobQ(3)	int_hyp(2)	omp25(8)
**ERZ4251209**	2	brucella	8	gap(3)	aroA(2)	glk(3)	dnaK(2)	gyrB(1)	trpE(5)	cobQ(3)	int_hyp(2)	omp25(8)
**ERZ4251210**	2	brucella	8	gap(3)	aroA(2)	glk(3)	dnaK(2)	gyrB(1)	trpE(5)	cobQ(3)	int_hyp(2)	omp25(8)
**ERZ4251211**	2	brucella	8	gap(3)	aroA(2)	glk(3)	dnaK(2)	gyrB(1)	trpE(5)	cobQ(3)	int_hyp(2)	omp25(8)
**ERZ4251212**	2	brucella	8	gap(3)	aroA(2)	glk(3)	dnaK(2)	gyrB(1)	trpE(5)	cobQ(3)	int_hyp(2)	omp25(8)
**SRR11178063**	1	brucella	8	gap(3)	aroA(2)	glk(3)	dnaK(2)	gyrB(1)	trpE(5)	cobQ(3)	int_hyp(2)	omp25(8)
**SRR11178065**	1	brucella	8	gap(3)	aroA(2)	glk(3)	dnaK(2)	gyrB(1)	trpE(5)	cobQ(3)	int_hyp(2)	omp25(8)
**SRR11178066**	1	brucella	8	gap(3)	aroA(2)	glk(3)	dnaK(2)	gyrB(1)	trpE(5)	cobQ(3)	int_hyp(2)	omp25(8)
**SRR11178067**	1	brucella	8	gap(3)	aroA(2)	glk(3)	dnaK(2)	gyrB(1)	trpE(5)	cobQ(3)	int_hyp(2)	omp25(8)
**SRR11178068**	1	brucella	8	gap(3)	aroA(2)	glk(3)	dnaK(2)	gyrB(1)	trpE(5)	cobQ(3)	int_hyp(2)	omp25(8)
**SRR11178069**	1	brucella	8	gap(3)	aroA(2)	glk(3)	dnaK(2)	gyrB(1)	trpE(5)	cobQ(3)	int_hyp(2)	omp25(8)
**SRR11178070**	1	brucella	8	gap(3)	aroA(2)	glk(3)	dnaK(2)	gyrB(1)	trpE(5)	cobQ(3)	int_hyp(2)	omp25(8)
**SRR11178071**	1	brucella	8	gap(3)	aroA(2)	glk(3)	dnaK(2)	gyrB(1)	trpE(5)	cobQ(3)	int_hyp(2)	omp25(8)
**SRR11178072**	1	brucella	8	gap(3)	aroA(2)	glk(3)	dnaK(2)	gyrB(1)	trpE(5)	cobQ(3)	int_hyp(2)	omp25(8)
**SRR11178073**	1	brucella	8	gap(3)	aroA(2)	glk(3)	dnaK(2)	gyrB(1)	trpE(5)	cobQ(3)	int_hyp(2)	omp25(8)
**SRR11178074**	1	brucella	8	gap(3)	aroA(2)	glk(3)	dnaK(2)	gyrB(1)	trpE(5)	cobQ(3)	int_hyp(2)	omp25(8)
**SRR11178075**	1	brucella	8	gap(3)	aroA(2)	glk(3)	dnaK(2)	gyrB(1)	trpE(5)	cobQ(3)	int_hyp(2)	omp25(8)
**SRR11178076**	1	brucella	8	gap(3)	aroA(2)	glk(3)	dnaK(2)	gyrB(1)	trpE(5)	cobQ(3)	int_hyp(2)	omp25(8)
**SRR11178077**	1	brucella	8	gap(3)	aroA(2)	glk(3)	dnaK(2)	gyrB(1)	trpE(5)	cobQ(3)	int_hyp(2)	omp25(8)
**SRR11178078**	1	brucella	8	gap(3)	aroA(2)	glk(3)	dnaK(2)	gyrB(1)	trpE(5)	cobQ(3)	int_hyp(2)	omp25(8)

**SRR11178079**	1	brucella	8	gap(3)	aroA(2)	glk(3)	dnaK(2)	gyrB(1)	trpE(5)	cobQ(3)	int_hyp(2)	omp25(8)
**SRR11178080**	1	brucella	8	gap(3)	aroA(2)	glk(3)	dnaK(2)	gyrB(1)	trpE(5)	cobQ(3)	int_hyp(2)	omp25(8)
**SRR11178081**	1	brucella	8	gap(3)	aroA(2)	glk(3)	dnaK(2)	gyrB(1)	trpE(5)	cobQ(3)	int_hyp(2)	omp25(8)
**SRR11178083**	1	brucella	8	gap(3)	aroA(2)	glk(3)	dnaK(2)	gyrB(1)	trpE(5)	cobQ(3)	int_hyp(2)	omp25(8)
**SRR11178084**	1	brucella	8	gap(3)	aroA(2)	glk(3)	dnaK(2)	gyrB(1)	trpE(5)	cobQ(3)	int_hyp(2)	omp25(8)
**SRR11178085**	1	brucella	8	gap(3)	aroA(2)	glk(3)	dnaK(2)	gyrB(1)	trpE(5)	cobQ(3)	int_hyp(2)	omp25(8)
**SRR11178086**	1	brucella	8	gap(3)	aroA(2)	glk(3)	dnaK(2)	gyrB(1)	trpE(5)	cobQ(3)	int_hyp(2)	omp25(8)
**SRR11178087**	1	brucella	8	gap(3)	aroA(2)	glk(3)	dnaK(2)	gyrB(1)	trpE(5)	cobQ(3)	int_hyp(2)	omp25(8)
**SRR11178088**	1	brucella	8	gap(3)	aroA(2)	glk(3)	dnaK(2)	gyrB(1)	trpE(5)	cobQ(3)	int_hyp(2)	omp25(8)
**SRR11178089**	1	brucella	8	gap(3)	aroA(2)	glk(3)	dnaK(2)	gyrB(1)	trpE(5)	cobQ(3)	int_hyp(2)	omp25(8)

References

**1**	Rabinowitz, Peter, et al. “Whole Genome Sequence Analysis of Brucella Melitensis Phylogeny and Virulence Factors.” Microbiology Research, vol. 12, no. 3, Aug. 2021, pp. 698–710. DOI.org (Crossref), https://doi.org/10.3390/microbiolres12030050.
**2**	Zilberman, Bar, et al. “Genomic Epidemiology of Clinical Brucella Melitensis Isolates from Southern Israel.” Microorganisms, vol. 10, no. 2, Jan. 2022, p. 238. DOI.org (Crossref), https://doi.org/10.3390/microorganisms10020238.

Table S4. General dairy farm information

Farm code	Geographic area	Size	Notes
**1**	South	Large	
**2**	South	Medium	
**3**	South	No data	
**4**	South	Small	
**5**	South	Small	
**6**	South	Small	
**7**	Center	Small	
**8**	North	Small	
**9**	North	Small	
**10**	South	Large	
**11**	South	Medium	
**12**	South	Medium	
**13**	South	Medium	
**14**	North	Medium	
**15**	South	Medium	
**16**	South	Medium	

**17**	South	Medium	
**18**	North	Small	
**19**	North	Medium	
**20**	South	Not applicable	Other form of animal husbandry
**21**	South	Not applicable	
**22**	North	Not applicable	
**23**	North	Not applicable	

Table S5. Accession numbers for sequenced isolates

Study ID	Study Bioproject ID	Sample Experiment ID	Sample ID	Reads Run ID	Assembly Result ID
**B2**	PRJEB52526	ERX9970074	ERS13635777	ERR10445441	ERZ14230476
**B5**	PRJEB52526	ERX9970090	ERS13635795	ERR10445457	ERZ14230494
**B6**	PRJEB52526	ERX9970099	ERS13635801	ERR10445466	ERZ14230500
**B7**	PRJEB52526	ERX9970105	ERS13635809	ERR10445472	ERZ14230508
**B8**	PRJEB52526	ERX9970113	ERS13635814	ERR10445480	ERZ14230513
**B9**	PRJEB52526	ERX9970118	ERS13635815	ERR10445485	ERZ14230514
**B10**	PRJEB52526	ERX9970064	ERS13635710	ERR10445431	ERZ14230409
**B11**	PRJEB52526	ERX9970065	ERS13635721	ERR10445432	ERZ14230420
**B12**	PRJEB52526	ERX9970066	ERS13635732	ERR10445433	ERZ14230431
**B13**	PRJEB52526	ERX9970067	ERS13635742	ERR10445434	ERZ14230441
**B14**	PRJEB52526	ERX9970068	ERS13635752	ERR10445435	ERZ14230451
**B15**	PRJEB52526	ERX9970069	ERS13635763	ERR10445436	ERZ14230462
**B16**	PRJEB52526	ERX9970070	ERS13635767	ERR10445437	ERZ14230466
**B17**	PRJEB52526	ERX9970071	ERS13635768	ERR10445438	ERZ14230467
**B18**	PRJEB52526	ERX9970072	ERS13635769	ERR10445439	ERZ14230468
**B19**	PRJEB52526	ERX9970073	ERS13635770	ERR10445440	ERZ14230469
**B20**	PRJEB52526	ERX9970075	ERS13635771	ERR10445442	ERZ14230470
**B21**	PRJEB52526	ERX9970076	ERS13635772	ERR10445443	ERZ14230471
**B22**	PRJEB52526	ERX9970077	ERS13635773	ERR10445444	ERZ14230472
**B23**	PRJEB52526	ERX9970078	ERS13635774	ERR10445445	ERZ14230473
**B24**	PRJEB52526	ERX9970079	ERS13635775	ERR10445446	ERZ14230474
**B25**	PRJEB52526	ERX9970080	ERS13635776	ERR10445447	ERZ14230475
**B32**	PRJEB52526	ERX9970081	ERS13635778	ERR10445448	ERZ14230477
**B35**	PRJEB52526	ERX9970082	ERS13635779	ERR10445449	ERZ14230478
**B36**	PRJEB52526	ERX9970083	ERS13635780	ERR10445450	ERZ14230479
**B37**	PRJEB52526	ERX9970084	ERS13635781	ERR10445451	ERZ14230480
**B38**	PRJEB52526	ERX9970085	ERS13635782	ERR10445452	ERZ14230481
**B39**	PRJEB52526	ERX9970086	ERS13635783	ERR10445453	ERZ14230482

**B40**	PRJEB52526	ERX9970087	ERS13635784	ERR10445454	ERZ14230483
**B47**	PRJEB52526	ERX9970088	ERS13635785	ERR10445455	ERZ14230484
**B48**	PRJEB52526	ERX9970089	ERS13635786	ERR10445456	ERZ14230485
**B50**	PRJEB52526	ERX9970091	ERS13635787	ERR10445458	ERZ14230486
**B51**	PRJEB52526	ERX9970092	ERS13635788	ERR10445459	ERZ14230487
**B52**	PRJEB52526	ERX9970093	ERS13635789	ERR10445460	ERZ14230488
**B53**	PRJEB52526	ERX9970094	ERS13635790	ERR10445461	ERZ14230489
**B54**	PRJEB52526	ERX9970095	ERS13635791	ERR10445462	ERZ14230490
**B55**	PRJEB52526	ERX9970096	ERS13635792	ERR10445463	ERZ14230491
**B56**	PRJEB52526	ERX9970097	ERS13635793	ERR10445464	ERZ14230492
**B57**	PRJEB52526	ERX9970098	ERS13635794	ERR10445465	ERZ14230493
**B65**	PRJEB52526	ERX9970100	ERS13635796	ERR10445467	ERZ14230495
**B66**	PRJEB52526	ERX9970101	ERS13635797	ERR10445468	ERZ14230496
**B67**	PRJEB52526	ERX9970102	ERS13635798	ERR10445469	ERZ14230497
**B68**	PRJEB52526	ERX9970103	ERS13635799	ERR10445470	ERZ14230498
**B69**	PRJEB52526	ERX9970104	ERS13635800	ERR10445471	ERZ14230499
**B70**	PRJEB52526	ERX9970106	ERS13635802	ERR10445473	ERZ14230501
**B71**	PRJEB52526	ERX9970107	ERS13635803	ERR10445474	ERZ14230502
**B75**	PRJEB52526	ERX9970108	ERS13635804	ERR10445475	ERZ14230503
**B76**	PRJEB52526	ERX9970109	ERS13635805	ERR10445476	ERZ14230504
**B77**	PRJEB52526	ERX9970110	ERS13635806	ERR10445477	ERZ14230505
**B78**	PRJEB52526	ERX9970111	ERS13635807	ERR10445478	ERZ14230506
**B79**	PRJEB52526	ERX9970112	ERS13635808	ERR10445479	ERZ14230507
**B80**	PRJEB52526	ERX9970114	ERS13635810	ERR10445481	ERZ14230509
**B81**	PRJEB52526	ERX9970115	ERS13635811	ERR10445482	ERZ14230510
**B82**	PRJEB52526	ERX9970116	ERS13635812	ERR10445483	ERZ14230511
**B85**	PRJEB52526	ERX9970117	ERS13635813	ERR10445484	ERZ14230512
**B103**	PRJEB52526	ERX9970119	ERS13635704	ERR10445486	ERZ14230403
**B104**	PRJEB52526	ERX9970120	ERS13635705	ERR10445487	ERZ14230404
**B106**	PRJEB52526	ERX9970121	ERS13635706	ERR10445488	ERZ14230405
**B107**	PRJEB52526	ERX9970122	ERS13635707	ERR10445489	ERZ14230406
**B108**	PRJEB52526	ERX9970123	ERS13635708	ERR10445490	ERZ14230407

**B109**	PRJEB52526	ERX9970124	ERS13635709	ERR10445491	ERZ14230408
**B110**	PRJEB52526	ERX9970125	ERS13635711	ERR10445492	ERZ14230410
**B111**	PRJEB52526	ERX9970126	ERS13635712	ERR10445493	ERZ14230411
**B112**	PRJEB52526	ERX9970127	ERS13635713	ERR10445494	ERZ14230412
**B113**	PRJEB52526	ERX9970128	ERS13635714	ERR10445495	ERZ14230413
**B114**	PRJEB52526	ERX9970129	ERS13635715	ERR10445496	ERZ14230414
**B115**	PRJEB52526	ERX9970130	ERS13635716	ERR10445497	ERZ14230415
**B116**	PRJEB52526	ERX9970131	ERS13635717	ERR10445498	ERZ14230416
**B117**	PRJEB52526	ERX9970132	ERS13635718	ERR10445499	ERZ14230417
**B118**	PRJEB52526	ERX9970133	ERS13635719	ERR10445500	ERZ14230418
**B119**	PRJEB52526	ERX9970134	ERS13635720	ERR10445501	ERZ14230419
**B120**	PRJEB52526	ERX9970135	ERS13635722	ERR10445502	ERZ14230421
**B121**	PRJEB52526	ERX9970136	ERS13635723	ERR10445503	ERZ14230422
**B122**	PRJEB52526	ERX9970137	ERS13635724	ERR10445504	ERZ14230423
**B123**	PRJEB52526	ERX9970138	ERS13635725	ERR10445505	ERZ14230424
**B124**	PRJEB52526	ERX9970139	ERS13635726	ERR10445506	ERZ14230425
**B125**	PRJEB52526	ERX9970140	ERS13635727	ERR10445507	ERZ14230426
**B126**	PRJEB52526	ERX9970141	ERS13635728	ERR10445508	ERZ14230427
**B127**	PRJEB52526	ERX9970142	ERS13635729	ERR10445509	ERZ14230428
**B128**	PRJEB52526	ERX9970144	ERS13635730	ERR10445511	ERZ14230429
**B129**	PRJEB52526	ERX9970146	ERS13635731	ERR10445513	ERZ14230430
**B130**	PRJEB52526	ERX9970147	ERS13635733	ERR10445514	ERZ14230432
**B131**	PRJEB52526	ERX9970148	ERS13635734	ERR10445515	ERZ14230433
**B132**	PRJEB52526	ERX9970149	ERS13635735	ERR10445516	ERZ14230434
**B133**	PRJEB52526	ERX9970150	ERS13635736	ERR10445517	ERZ14230435
**B134**	PRJEB52526	ERX9970151	ERS13635737	ERR10445518	ERZ14230436
**B135**	PRJEB52526	ERX9970152	ERS13635738	ERR10445519	ERZ14230437
**B137**	PRJEB52526	ERX9970153	ERS13635739	ERR10445520	ERZ14230438
**B138**	PRJEB52526	ERX9970154	ERS13635740	ERR10445521	ERZ14230439
**B139**	PRJEB52526	ERX9970155	ERS13635741	ERR10445522	ERZ14230440
**B140**	PRJEB52526	ERX9970156	ERS13635743	ERR10445523	ERZ14230442
**B141**	PRJEB52526	ERX9970158	ERS13635744	ERR10445525	ERZ14230443

**B142**	PRJEB52526	ERX9970159	ERS13635745	ERR10445526	ERZ14230444
**B143**	PRJEB52526	ERX9970160	ERS13635746	ERR10445527	ERZ14230445
**B144**	PRJEB52526	ERX9970161	ERS13635747	ERR10445528	ERZ14230446
**B145**	PRJEB52526	ERX9970162	ERS13635748	ERR10445529	ERZ14230447
**B146**	PRJEB52526	ERX9970163	ERS13635749	ERR10445530	ERZ14230448
**B148**	PRJEB52526	ERX9970164	ERS13635750	ERR10445531	ERZ14230449
**B149**	PRJEB52526	ERX9970165	ERS13635751	ERR10445532	ERZ14230450
**B150**	PRJEB52526	ERX9970166	ERS13635753	ERR10445533	ERZ14230452
**B151**	PRJEB52526	ERX9970167	ERS13635754	ERR10445534	ERZ14230453
**B152**	PRJEB52526	ERX9970168	ERS13635755	ERR10445535	ERZ14230454
**B153**	PRJEB52526	ERX9970169	ERS13635756	ERR10445536	ERZ14230455
**B154**	PRJEB52526	ERX9970170	ERS13635757	ERR10445537	ERZ14230456
**B155**	PRJEB52526	ERX9970171	ERS13635758	ERR10445538	ERZ14230457
**B156**	PRJEB52526	ERX9970172	ERS13635759	ERR10445539	ERZ14230458
**B157**	PRJEB52526	ERX9970173	ERS13635760	ERR10445540	ERZ14230459
**B158**	PRJEB52526	ERX9970174	ERS13635761	ERR10445541	ERZ14230460
**B159**	PRJEB52526	ERX9970175	ERS13635762	ERR10445542	ERZ14230461
**B160**	PRJEB52526	ERX9970176	ERS13635764	ERR10445543	ERZ14230463
**B161**	PRJEB52526	ERX9970177	ERS13635765	ERR10445544	ERZ14230464
**B162**	PRJEB52526	ERX9970178	ERS13635766	ERR10445545	ERZ14230465

Figure S1. Reference genome selection.

Neighbour-Joining tree generated from Mashtree of 112 genome assemblies from this study (dark blue nodes) and 70 RefSeq *B. melitensis* complete genomes (light blue nodes). RefSeq genomes selected for the SNPs tree analysis are highlighted in red. Reference genomes downloaded and analysed and those selected for inclusion in the SNP tree are listed below.

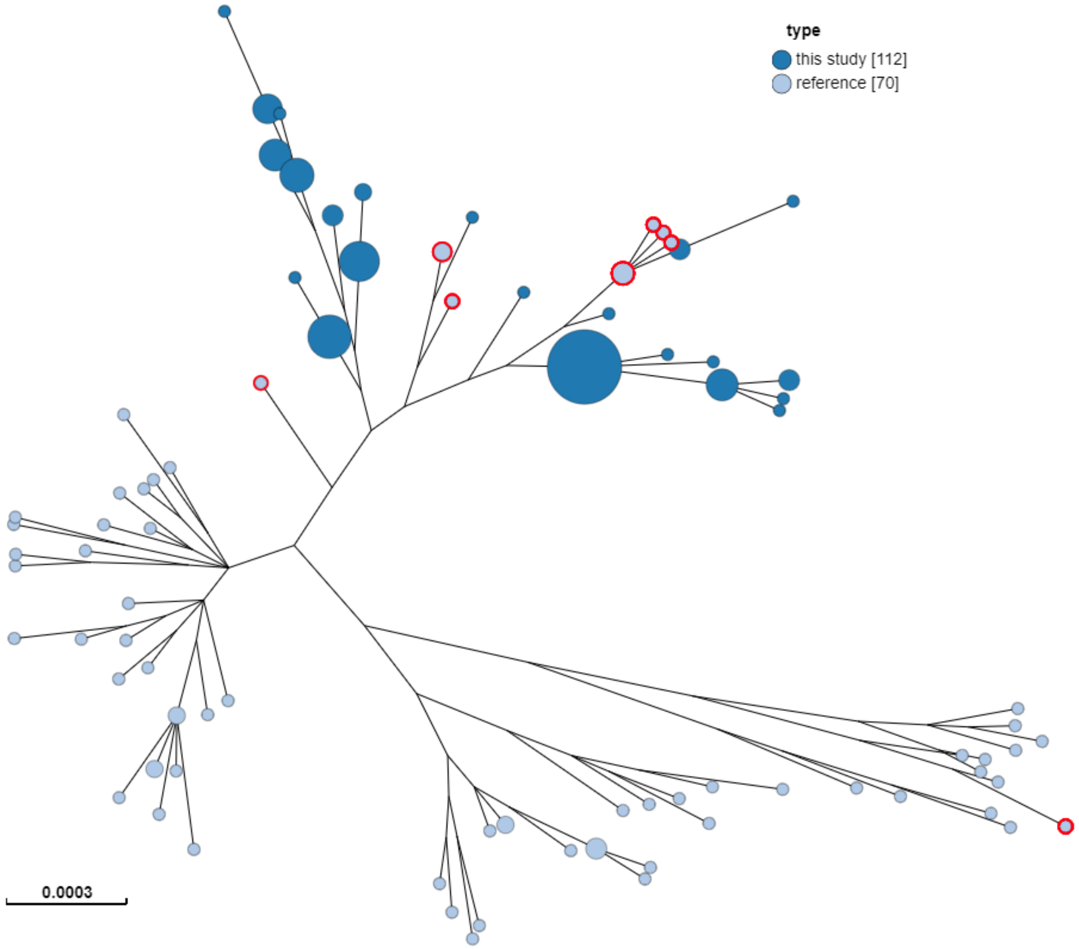

***B. melitensis* reference genomes downloaded from NCBI RefSeq database (n=70) and those selected for inclusion in the SNP tree (in red font).**

GCF_000007125.1	GCF_002191375.1	GCF_002895105.1	GCF_009017395.1	GCF_900236435.1
GCF_000022625.1	GCF_002191455.1	GCF_002895125.1	GCF_023796775.1	GCF_900236445.1
GCF_000192725.1	GCF_002191575.1	GCF_002953595.1	GCF_024802385.1	GCF_900236455.1
GCF_000192885.1	GCF_002191615.1	GCF_003205535.1	GCF_027625455.1	GCF_900236465.1
GCF_000227645.1	GCF_002191655.1	GCF_003516045.1	GCF_900236335.1	GCF_900236485.1
GCF_000740355.1	GCF_002191675.1	GCF_003516065.1	GCF_900236345.1	GCF_900236495.1
GCF_000740415.1	GCF_002191755.1	GCF_003516085.1	GCF_900236355.1	GCF_900236515.1
GCF_001307475.2	GCF_002191915.1	GCF_003856415.1	GCF_900236365.1	GCF_900236525.1
GCF_001431745.1	GCF_002192095.1	GCF_004208655.1	GCF_900236375.1	GCF_900236535.1
GCF_001715485.1	GCF_002192155.1	GCF_004208675.1	GCF_900236385.1	GCF_900236545.1
GCF_002191235.1	GCF_002214285.1	GCF_004208695.1	GCF_900236395.1	GCF_900236555.1
GCF_002191295.1	GCF_002262955.1	GCF_008761595.1	GCF_900236405.1	GCF_900236565.1
GCF_002191335.1	GCF_002263015.1	GCF_008761615.1	GCF_900236415.1	GCF_900236575.1
GCF_002191355.1	GCF_002763615.1	GCF_009017355.1	GCF_900236425.1	GCF_900236585.1

GCF_002191335.1
GCF_002191455.1
GCF_002191615.1
GCF_002191655.1
GCF_002191675.1
GCF_002191755.1
GCF_002192095.1
GCF_002192155.1
GCF_003856415.1

Table S6. Results of pan-genome analysis of bovine vs. non-bovine isolates from Southern Israel.

Annotation	Bovine (n=60)	non-Bovine (n=133)	P value
hypothetical protein	13%	53%	1.73E-05
hypothetical protein	17%	56%	1.73E-05
hypothetical protein	17%	56%	1.73E-05
hypothetical protein	17%	56%	1.73E-05
hypothetical protein	17%	56%	1.73E-05
hypothetical protein	17%	56%	1.73E-05
IS5 family transposase	80%	43%	0.00018
IS6-like element IS2020 family transposase	20%	1%	0.000288
hypothetical protein	5%	32%	0.001258
IS5 family transposase	35%	9%	0.001526
tRNA guanosine(34) transglycosylase Tgt	30%	8%	0.018279
hypothetical protein	12%	35%	0.046445

Figure S2. Phylogenetic analysis of the bovine cohort using core genome SNP analysis.

Phylogenetic analysis6 of the bovine-human isolate cohort (n=92) based on an *cgSNP* analysis. Colour coding denotes the investigated epidemiological clusters. Node size is proportional to the number of isolates assigned to clone types. Numbers denote the SNP distances between nodes. Outlines define genomically related isolates (genomic clusters).

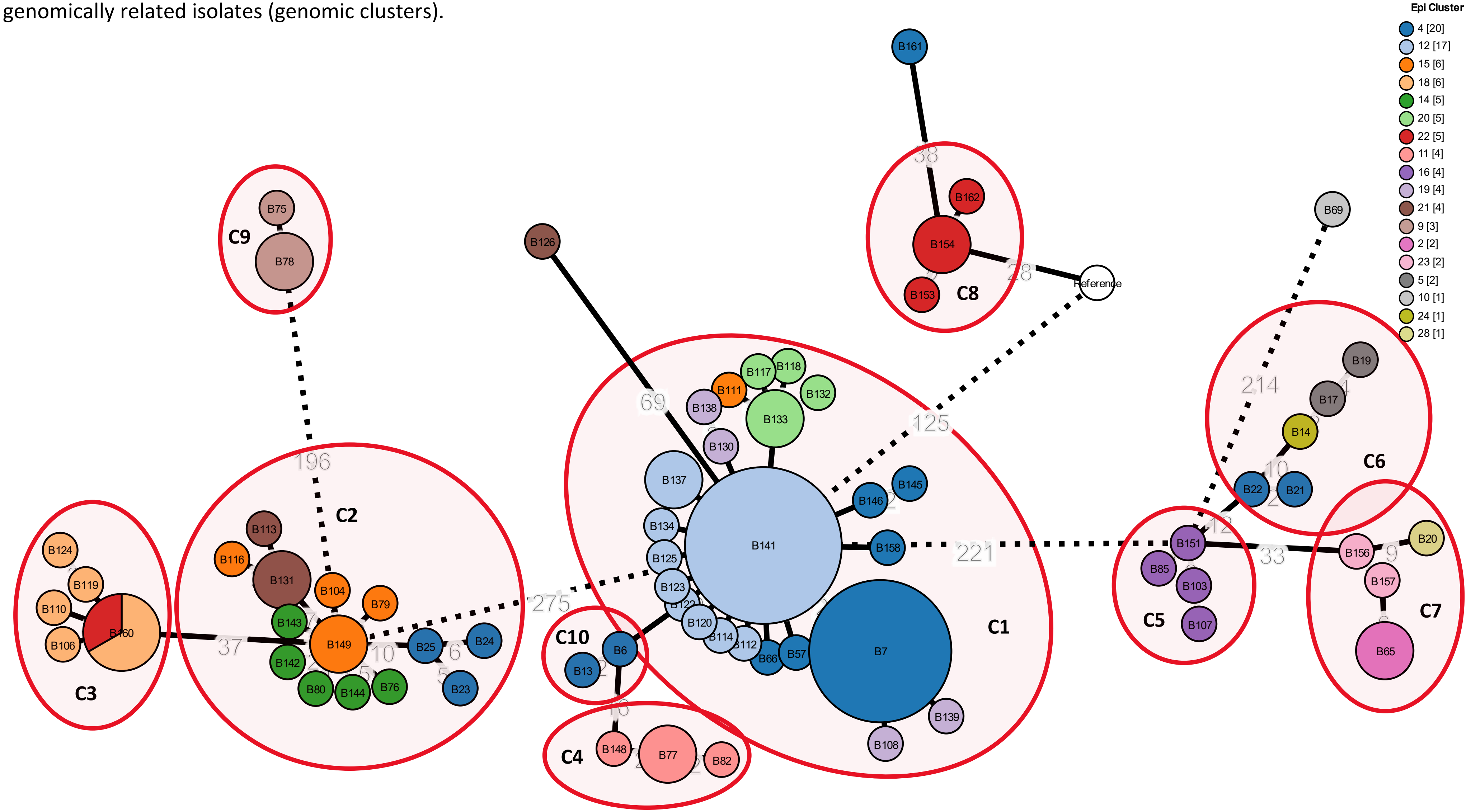

Supplement: Supplementary material 1 [file mgen-9-1014-s001.pdf]
